# Acclimatize experimental approach to adjudicate hydraulic coefficients under different bed material configurations and slopes with and without weir

**DOI:** 10.1016/j.heliyon.2024.e32162

**Published:** 2024-06-01

**Authors:** Ayalkie Belete Amsie, Abebe Temesgen Ayalew, Zerihun Makayno Mada, Mekuanent Muluneh Finsa

**Affiliations:** a Water Resources Research Center, Arba Minch University, Ethiopia; b Faculty of Hydraulic and Water Resources Engineering, Arba Minch University, Arba Minch, Ethiopia

**Keywords:** Manning's and discharge coefficient, Bed roughness, Rectangular weir bed distribution, Froude number, Reynolds number, Open channel flow

## Abstract

The primary purpose of this study was to evaluate the hydraulic coefficient of coarse aggregate grain size beds and hydraulic parameters under random and perpendicular bed configurations, as well as to explore the discharge coefficient for rectangular weirs. The research objectives were to compare flow resistance coefficients, evaluate the discharge coefficient for rectangular weirs, investigate the relationship between roughness coefficient and hydraulic parameters, and validate the theoretical hydraulic equation for the rectangular weir. This was achieved by analysing different bed configurations, bed slopes, and 20 and 30-mm bed materials. Sieve analysis was conducted on bed materials using American-standard sieves to determine their particle size distribution. The experiment was performed in a rectangular flume measuring 12 m in length, 0.31 m in width, and 0.45 m in depth. In a laboratory experiment, water was pumped into a flume using centrifugal pumps, and a rectangular weir was attached downstream for discharge measurement. The experiment investigated factors such as Manning roughness coefficient, bed material geometry, bed slope, and weir shapes. Approximately 1680 tests were conducted to analysed the impact of these factors on discharge and the coefficient of discharge. The average Manning's roughness coefficients for a grain size of 20 mm were 0.019 (with weir) and 0.019 (without weir) in a random bed configuration, and 0.028 (with weir) and 0.027 (without weir) in a perpendicular flow bed configuration. For a grain size of 30 mm, the coefficients were 0.023 (with weir) and 0.022 (without weir) in a random bed configuration, and 0.033 (with weir) and 0.026 (without weir) in a perpendicular flow bed configuration. The presence of a weir has affected Manning's roughness coefficients and discharge coefficients. With a weir, the roughness coefficients have generally been higher compared to without a weir, indicating increased roughness in the channel. The discharge coefficient for a rectangular weir with a grain size of 20 mm ranged from 0.39 to 0.84 (random bed) and 0.27 to 0.68 (perpendicular flow bed), while for a grain size of 30 mm it ranged from 0.31 to 0.81 (random bed) and 0.23 to 0.48 (perpendicular flow bed). The discharge coefficients have varied depending on the grain size and bed configuration, reflecting different flow efficiencies over the weir. Rough particles influenced flow and Manning's roughness coefficient value, then reduced discharge and velocity values. Under two bed configurations and slopes, beds with a grain size of 30 mm have higher roughness coefficients compared to those with a grain size of 20 mm. The models have shown that the roughness coefficient is inversely proportional to the discharge and directly proportional to the tailgate water levels. The coefficient of roughness and discharge coefficient are mainly influenced by the channel slopes, bed roughness, bed grain size, and bed configuration. A randomly configured bed with a 20 mm grain size gravel bed is preferred over a perpendicular bed configuration to handle high discharges. Using a 20 mm grain-size gravel bed in open-channel flow is more suitable than a 30 mm grain-size bed. Relying on the constant friction factor, Manning's n, is not recommended as it may result in design errors. These findings have the potential to improve hydraulic engineering design practices, enhancing the accuracy and efficiency of open-channel flow systems.

## 1 Introduction

Hydraulic coefficients play a critical role in the design and analysis of open channel flow systems. They are used to characterize the behaviour of water in channels and provide important information for designing and managing various water structures [1]. These coefficients are essential for designing and optimizing the performance of water structures such as canals, sewers, and dams. By considering the hydraulic coefficients, engineers can ensure that the flow characteristics and energy losses in these structures are within acceptable limits, leading to efficient and safe operation [2,3]. Dam design, irrigation and drainage network planning, flood simulation, sediment transport, urban water supplies are considered on flow measurements and computed hydraulic coefficient ([1]; Ahmed et al., 2017; [4]). The Manning roughness coefficient (n) is a key parameter in open channel flow analysis and design. It represents the resistance to flow caused by the channel's roughness characteristics, including vegetation, channel shape, and surface roughness (Chen et al., 2009; [2,5]). Cross section area, wetted perimeter, hydraulic radius, Froude number, depth of flow, bed slope of water surface, discharge coefficient, bed surfaces and bed pattern, Chezy's constant, and coefficient of roughness are considered as important factors that affect velocity and discharge in a given channel ([6]; Hari et al., 2015; [7]). ‬‬‬‬‬‬‬‬‬‬‬‬‬‬‬‬‬‬‬‬‬‬‬‬‬‬‬‬‬‬‬‬‬‬‬‬‬‬‬‬‬‬‬‬‬‬‬‬‬‬‬‬‬‬‬‬‬‬‬‬‬‬‬‬‬‬‬‬‬‬‬‬‬‬‬‬‬‬‬‬‬‬‬‬‬‬‬‬‬‬‬‬‬‬‬‬‬‬‬‬‬‬‬‬‬‬‬‬‬‬‬‬‬‬‬‬‬‬‬‬‬‬‬‬‬‬‬‬‬‬‬‬‬‬‬‬‬‬‬‬‬‬‬‬‬‬‬‬‬‬‬‬‬‬‬‬‬‬‬‬‬‬‬‬‬‬‬‬‬‬‬‬‬‬‬‬‬‬‬‬‬‬‬‬‬‬‬‬‬‬‬‬‬‬‬‬‬‬‬‬‬‬‬‬‬‬‬‬‬‬‬‬‬‬‬‬‬‬‬‬‬‬‬‬‬‬‬‬‬‬‬‬‬‬‬‬‬‬‬‬‬‬‬‬‬‬‬‬‬‬‬‬‬‬‬‬‬‬‬‬‬‬‬‬‬‬‬‬‬‬‬‬‬‬‬‬‬‬‬‬‬‬‬‬‬‬‬‬‬‬‬‬‬‬‬‬‬‬‬‬‬‬‬‬‬‬‬‬‬‬‬‬‬‬‬‬‬‬‬‬‬‬‬‬‬‬‬‬‬‬‬‬‬‬‬‬‬‬‬‬‬‬‬‬‬‬‬‬‬‬‬‬‬‬‬‬‬‬‬‬‬‬‬‬‬‬‬‬‬‬‬‬‬‬‬‬‬‬‬‬‬‬‬‬‬‬‬‬‬‬‬‬‬‬‬‬‬‬‬‬‬‬‬‬‬‬‬‬‬‬‬‬‬‬‬‬‬‬‬‬‬‬‬‬‬‬‬‬‬‬‬‬‬‬‬‬‬‬‬‬‬‬‬‬‬‬‬‬‬‬‬‬‬‬‬‬‬‬‬‬‬‬‬‬‬‬‬‬‬‬‬‬‬‬‬‬‬‬‬‬‬‬‬‬‬‬‬‬‬‬‬‬‬‬‬‬‬‬‬‬‬‬‬‬‬‬‬‬‬‬‬‬‬‬‬‬‬‬‬‬‬‬‬‬‬‬‬‬‬‬‬‬‬‬‬‬‬‬‬‬‬‬‬‬‬‬‬‬‬‬‬‬‬‬‬‬‬‬‬‬‬‬‬‬‬‬‬‬‬‬‬‬‬‬‬‬‬‬‬‬‬‬‬‬‬‬‬‬‬‬‬‬‬‬‬‬‬‬‬‬‬‬‬‬‬‬‬‬‬‬‬‬‬‬‬‬‬‬‬‬‬‬‬‬‬‬‬‬‬‬‬‬‬‬‬‬‬‬‬‬‬‬‬‬‬‬‬‬‬‬‬‬‬‬‬‬‬‬‬‬‬‬‬‬‬‬‬‬‬‬‬‬‬‬‬‬‬‬‬‬‬‬‬‬‬‬‬‬‬‬‬‬‬‬‬‬‬‬‬‬‬‬‬‬‬‬‬‬‬‬‬‬‬‬‬‬‬‬‬‬‬‬‬‬‬‬‬‬‬‬‬‬‬‬‬‬‬‬‬‬‬‬‬‬‬‬‬‬‬‬‬‬‬‬‬‬‬‬‬‬‬‬‬‬‬‬‬‬‬‬‬‬‬‬‬‬‬‬‬‬‬‬‬‬‬‬‬‬‬‬‬‬‬‬‬‬‬‬‬‬‬‬‬‬‬‬‬‬‬‬‬‬‬‬‬‬‬‬‬‬‬‬‬‬‬‬‬‬‬‬‬‬‬‬‬‬‬‬‬‬‬‬‬‬‬‬‬‬‬‬‬‬‬‬‬‬‬‬‬‬‬‬‬‬‬‬‬‬‬‬‬‬‬‬‬‬‬‬‬‬‬‬‬‬‬‬‬‬‬‬‬‬‬‬‬‬‬‬‬‬‬‬‬‬‬‬‬‬‬‬‬‬‬‬‬‬‬‬‬‬‬‬‬‬‬‬‬‬‬‬‬‬‬‬‬‬‬‬‬‬‬‬‬‬‬‬‬‬‬‬‬‬‬‬‬‬‬‬‬‬‬‬‬‬‬‬‬‬‬‬‬‬‬‬‬‬‬‬‬‬‬‬‬‬‬‬‬‬‬‬‬‬‬‬‬‬‬‬‬‬‬‬‬‬‬‬‬‬‬‬‬‬‬‬‬‬‬‬‬‬‬‬‬‬‬‬‬‬‬‬‬‬‬‬‬‬‬‬‬‬‬‬‬‬‬‬‬‬‬‬‬‬‬‬‬‬‬‬‬‬‬‬‬‬‬‬‬‬‬‬‬‬‬‬‬‬‬‬‬‬‬‬‬‬‬‬‬‬‬‬‬‬‬‬‬‬‬‬‬‬‬‬‬‬‬‬‬‬‬‬‬‬‬‬‬‬‬‬‬‬‬‬‬‬‬‬‬‬‬‬‬‬‬‬‬‬‬‬‬‬‬‬‬‬‬‬‬‬‬‬‬‬‬‬‬‬‬‬‬‬‬‬‬‬‬‬‬‬‬‬‬‬‬‬‬‬‬‬‬‬‬‬‬‬‬‬‬‬‬‬‬‬‬‬‬‬‬‬‬‬‬‬‬‬‬‬‬‬‬‬‬‬‬‬‬‬‬‬‬‬‬‬‬‬‬‬‬‬‬‬‬‬‬‬‬‬‬‬‬‬‬‬‬‬‬‬‬‬‬‬‬‬‬‬‬‬‬‬‬‬‬‬‬‬‬‬‬‬‬‬‬‬‬‬‬‬‬‬‬‬‬‬‬‬‬‬‬‬‬‬‬‬‬‬‬‬‬‬‬‬‬‬‬‬‬‬‬‬‬‬‬‬‬‬‬‬‬‬‬‬‬‬‬‬‬‬‬‬‬‬‬‬‬‬‬‬‬‬‬‬‬‬‬‬‬‬‬‬‬‬‬‬‬‬‬‬‬‬‬‬‬‬‬‬‬‬‬‬‬‬‬‬‬‬‬‬‬‬‬‬‬‬‬‬‬‬‬‬‬‬‬‬‬‬‬‬‬‬‬‬‬‬‬‬‬‬‬‬‬‬‬‬‬‬‬‬‬‬‬‬‬‬‬‬‬‬‬‬‬‬‬‬‬‬‬‬‬‬‬‬‬‬‬‬‬‬‬‬‬‬‬‬‬‬‬‬‬‬‬‬‬‬‬‬‬‬‬‬‬‬‬‬‬‬‬‬‬‬‬‬‬‬‬‬‬‬‬‬‬‬‬‬‬‬‬‬‬‬‬‬‬‬‬‬‬‬‬‬‬‬‬‬‬‬‬‬‬‬‬‬‬‬‬‬‬‬‬‬‬‬‬‬‬‬‬‬‬‬‬‬‬‬‬‬‬‬‬‬‬‬‬‬‬‬‬‬‬‬‬‬‬‬‬‬‬‬‬‬‬‬‬‬‬‬‬‬‬‬‬‬‬‬‬‬‬‬‬‬‬‬‬‬‬‬‬‬‬‬‬‬‬‬‬‬‬‬‬‬‬‬‬‬‬‬‬‬‬‬‬‬‬‬‬‬‬‬‬‬‬‬‬‬‬‬‬‬‬‬‬‬‬‬‬‬‬‬‬‬‬‬‬‬‬‬‬‬‬‬‬‬‬‬‬‬‬‬‬‬‬‬‬‬‬‬‬‬‬‬‬‬‬‬‬‬‬‬‬‬‬‬‬‬‬‬‬‬‬‬‬‬‬‬‬‬‬‬‬‬‬‬‬‬‬‬‬‬‬‬‬‬‬‬‬‬‬‬‬‬‬‬‬‬‬‬‬‬‬‬‬‬‬‬‬‬‬‬‬‬‬‬‬‬‬‬‬‬‬‬‬‬In open channel, the concept of uniform flow is the foundation for understanding and addressing most of the problems of water structures ([8]; Calmet and Jacques, 2013). Depending on the condition of the river channel, all water channels, from natural river beds to manmade river channels, exhibit unique roughness coefficients [[9], [10], [11], [12]]. ‬‬‬‬‬‬‬‬‬‬‬‬‬‬‬‬‬‬‬‬‬‬‬‬‬‬‬‬‬‬‬‬‬‬‬‬‬‬‬‬‬‬‬‬‬‬‬‬‬‬‬‬‬‬‬‬‬‬‬‬‬‬‬‬‬‬‬‬‬‬‬‬‬‬‬‬‬‬‬‬‬‬‬‬‬‬‬‬‬‬‬‬‬‬‬‬‬‬‬‬‬‬‬‬‬‬‬‬‬‬‬‬‬‬‬‬‬‬‬‬‬‬‬‬‬‬‬‬‬‬‬‬‬‬‬‬‬‬‬‬‬‬‬‬‬‬‬‬‬‬‬‬‬‬‬‬‬‬‬‬‬‬‬‬‬‬‬‬‬‬‬‬‬‬‬‬‬‬‬‬‬‬‬‬‬‬‬‬‬‬‬‬‬‬‬‬‬‬‬‬‬‬‬‬‬‬‬‬‬‬‬‬‬‬‬‬‬‬‬‬‬‬‬‬‬‬‬‬‬‬‬‬‬‬‬‬‬‬‬‬‬‬‬‬‬‬‬‬‬‬‬‬‬‬‬‬‬‬‬‬‬‬‬‬‬‬‬‬‬‬‬‬‬‬‬‬‬‬‬‬‬‬‬‬‬‬‬‬‬‬‬‬‬‬‬‬‬‬‬‬‬‬‬‬‬‬‬‬‬‬‬‬‬‬‬‬‬‬‬‬‬‬‬‬‬‬‬‬‬‬‬‬‬‬‬‬‬‬‬‬‬‬‬‬‬‬‬‬‬‬‬‬‬‬‬‬‬‬‬‬‬‬‬‬‬‬‬‬‬‬‬‬‬‬‬‬‬‬‬‬‬‬‬‬‬‬‬‬‬‬‬‬‬‬‬‬‬‬‬‬‬‬‬‬‬‬‬‬‬‬‬‬‬‬‬‬‬‬‬‬‬‬‬‬‬‬‬‬‬‬‬‬‬‬‬‬‬‬‬‬‬‬‬‬‬‬‬‬‬‬‬‬‬‬‬‬‬‬‬‬‬‬‬‬‬‬‬‬‬‬‬‬‬‬‬‬‬‬‬‬‬‬‬‬‬‬‬‬‬‬‬‬‬‬‬‬‬‬‬‬‬‬‬‬‬‬‬‬‬‬‬‬‬‬‬‬‬‬‬‬‬‬‬‬‬‬‬‬‬‬‬‬‬‬‬‬‬‬‬‬‬‬‬‬‬‬‬‬‬‬‬‬‬‬‬‬‬‬‬‬‬‬‬‬‬‬‬‬‬‬‬‬‬‬‬‬‬‬‬‬‬‬‬‬‬‬‬‬‬‬‬‬‬‬‬‬‬‬‬‬‬‬‬‬‬‬‬‬‬‬‬‬‬‬‬‬‬‬‬‬‬‬‬‬‬‬‬‬‬‬‬‬‬‬‬‬‬‬‬‬‬‬‬‬‬‬‬‬‬‬‬‬‬‬‬‬‬‬‬‬‬‬‬‬‬‬‬‬‬‬‬‬‬‬‬‬‬‬‬‬‬‬‬‬‬‬‬‬‬‬‬‬‬‬‬‬‬‬‬‬‬‬‬‬‬‬‬‬‬‬‬‬‬‬‬‬‬‬‬‬‬‬‬‬‬‬‬‬‬‬‬‬‬‬‬‬‬‬‬‬‬‬‬‬‬‬‬‬‬‬‬‬‬‬‬‬‬‬‬‬‬‬‬‬‬‬‬‬‬‬‬‬‬‬‬‬‬‬‬‬‬‬‬‬‬‬‬‬‬‬‬‬‬‬‬‬‬‬‬‬‬‬‬‬‬‬‬‬‬‬‬‬‬‬‬‬‬‬‬‬‬‬‬‬‬‬‬‬‬‬‬‬‬‬‬‬‬‬‬‬‬‬‬‬‬‬‬‬‬‬‬‬‬‬‬‬‬‬‬‬‬‬‬‬‬‬‬‬‬‬‬‬‬‬‬‬‬‬‬‬‬‬‬‬‬‬‬‬‬‬‬‬‬‬‬‬‬‬‬‬‬‬‬‬‬‬‬‬‬‬‬‬‬‬‬‬‬‬‬‬‬‬‬‬‬‬‬‬‬‬‬‬‬‬‬‬‬‬‬‬‬‬‬‬‬‬‬‬‬‬‬‬‬‬‬‬‬‬‬‬‬‬‬‬‬‬‬‬‬‬‬‬‬‬‬‬‬‬‬‬‬‬‬‬‬‬‬‬‬‬‬‬‬‬‬‬‬‬‬‬‬‬‬‬‬‬‬‬‬‬‬‬‬‬‬‬‬‬‬‬‬‬‬‬‬‬‬‬‬‬‬‬‬‬‬‬‬‬‬‬‬‬‬‬‬‬‬‬‬‬‬‬‬‬‬‬‬‬‬‬‬‬‬‬‬‬‬‬‬‬‬‬‬‬‬‬‬‬‬‬‬‬‬‬‬‬‬‬‬‬‬‬‬‬‬‬‬‬‬‬‬‬‬‬‬‬‬‬‬‬‬‬‬‬‬‬‬‬‬‬‬‬‬‬‬‬‬‬‬‬‬‬‬‬‬‬‬‬‬‬‬‬‬‬‬‬‬‬‬‬‬‬‬‬‬‬‬‬‬‬‬‬‬‬‬‬‬‬‬‬‬‬‬‬‬‬‬‬‬‬‬‬‬‬‬‬‬‬‬‬‬‬‬‬‬‬‬‬‬‬‬‬‬‬‬‬‬‬‬‬‬‬‬‬‬‬‬‬‬‬‬‬‬‬‬‬‬‬‬‬‬‬‬‬‬‬‬‬‬‬‬‬‬‬‬‬‬‬‬‬‬‬‬‬‬‬‬‬‬‬‬‬‬‬‬‬‬‬‬‬‬‬‬‬‬‬‬‬‬‬‬‬‬‬‬‬‬‬‬‬‬‬‬‬‬‬‬‬‬‬‬‬‬‬‬‬‬‬‬‬‬‬‬‬‬‬‬‬‬‬‬‬‬‬‬‬‬‬‬‬‬‬‬‬‬‬‬‬‬‬‬‬‬‬‬‬‬‬‬‬‬‬‬‬‬‬‬‬‬‬‬‬‬‬‬‬‬‬‬‬‬‬‬‬‬‬‬‬‬‬‬‬‬‬‬‬‬‬‬‬‬‬‬‬‬‬‬‬‬‬‬‬‬‬‬‬‬‬‬‬‬‬‬‬‬‬‬‬‬‬‬‬‬‬‬‬‬‬‬‬‬‬‬‬‬‬‬‬‬‬‬‬‬‬‬‬‬‬‬‬‬‬‬‬‬‬‬‬‬‬‬‬‬‬‬‬‬‬‬‬‬‬‬‬‬‬‬‬‬‬‬‬‬‬‬‬‬‬‬‬‬‬‬‬‬‬‬‬‬‬‬‬‬‬‬‬‬‬‬‬‬‬‬‬‬‬‬‬‬‬‬‬‬‬‬‬‬‬‬‬‬‬‬‬‬‬‬‬‬‬‬‬‬‬‬‬‬‬‬‬‬‬‬‬‬‬‬‬‬‬‬‬‬‬‬‬‬‬‬‬‬‬‬‬‬‬‬‬‬‬‬‬‬‬‬‬‬‬‬‬‬‬‬‬‬‬‬‬‬‬‬‬‬‬‬‬‬‬‬‬‬‬‬‬‬‬‬‬‬‬‬‬‬‬‬‬‬‬‬‬‬‬‬‬‬‬‬‬‬‬‬‬‬‬‬‬‬‬‬‬‬‬‬‬‬‬‬‬‬‬‬‬‬‬‬‬‬‬‬‬‬‬‬‬‬‬‬‬‬‬‬‬‬‬‬‬‬‬‬‬‬‬‬‬‬‬‬‬‬‬‬‬‬‬‬‬‬‬‬‬‬‬‬‬‬‬‬‬‬‬‬‬‬‬‬‬‬‬‬‬‬‬‬‬‬‬‬‬‬‬‬‬‬‬‬‬‬Multiple factors influence the Manning roughness coefficient, flow velocity, depth, discharge, and Froude number in water structures. These factors include stream geometry, boundary and surface roughness, bed materials and configuration, channel irregularities, sedimentation and erosion, blockage, channel size and shape, water level and flow, seasonal changes, suspended solids, and river bed load. For efficient and sustainable water structure design as well as good water management, these variables must be considered ([13]; Plakane et al., 2017; [4]). Numerous parameters, including canal wall roughness, bed slope, cross-sectional area, bed pattern, grain size of the bed material, water structure form, and bed material viscosity, affect the flow in an open channel [13,14]. Considering them is critical for accurately predicting and managing flow conditions in open channels and ensuring effective design and management of water structures ([10,15]; Valle and Gregory, 2006; Ye et al., 2008).

Flow measurement in open channels is one of the major concerns in hydraulic engineering. Weirs have been widely used in open waterways for flow measurement, flow diversion, and flow control [16]. Water supply, wastewater, and sewage systems rely on different types of notches. The ratio of actual discharge to theoretical discharge provided by the structure is known as the coefficient of discharge ([17]; Mohammed, 2017 [18]; Hossieni et al., 2018; [19]). Lot of researches has been carried out to determine the hydraulic coefficients of different materials under different bed slopes and bed materials [[20], [21], [22], [23]], but there are uncertainties regarding the influence of bottom slope, bed particle size, under various bed configuration and hydraulic coefficients, influence of particle shape on open channel flow and influence of bed slope on discharge, as well as the influence of roughness coefficient on discharge, Froude and Reynolds numbers, and the value of hydraulic coefficients in different bed materials under different channel slopes and bed configurations. A detailed laboratory investigation was carried out to estimate the discharge coefficient of rectangular notch with different bed materials, patterns, and bed slopes to determine hydraulic coefficients under different bed material configuration and slope with and without weir. In the development of the theoretical equation for this experimental research, this research assumed to describe the relationship between the Manning roughness and discharge coefficient, as well as hydraulic parameters and other important variables. The following requirements are under consideration. The observation eliminated the influences of turbulence and air entrainment, resistance of the flume's side wall was not included, the subsequent depth was taken as the temporal mean value of its changes throughout time, a one-dimensional steady flow was assumed, the channel was assumed to be straight, sloping, and rectangular, the fluid was assumed to have no compressible properties, the velocity distribution was assumed to be uniform in both the upstream and downstream sections, at the beginning and at the end of the jump, the pressure distribution was assumed to be hydrostatic. The existing research only focused on a man-made experimental channel and did not consider natural channels, the laboratory testing limited the direct applicability of the findings to natural channel flow behaviour, natural channel factors such as morphology, sediment dynamics, and vegetation were not considered in the laboratory study, the smooth glass walls used in the experimental channel do not represent the rough and irregular sidewalls of natural channels, the existing research used a limited range of bed materials, which may not reflect the diverse sediment compositions found in natural channels.

This study was stimulating and provided an in-depth examination of hydraulic coefficients in open channels while taking into consideration several various variables, including grain size, bed slopes, and bed layouts. It also examines how Manning's discharge and roughness coefficients are affected by the presence of rectangular weirs. This paper is organized into five sections: Introduction, Methodology, Results and Discussion, Recommendations, and Conclusion.

## 2 Materials and methods

### 2.1 Experimental setup

**Fig. 1:**
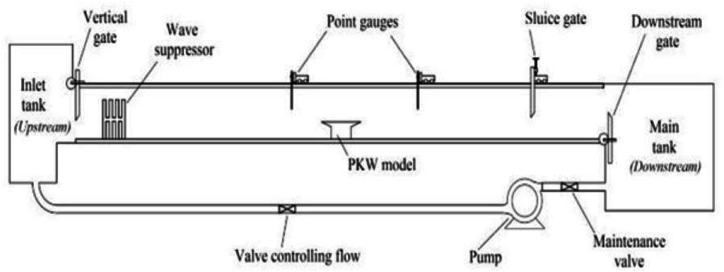
Plan View of Experimental Channel used in this study.

The experiments and computations conducted in the open channel laboratory flume have been completed in the laboratory of the Hydraulic and Water Resources Engineering Faculty, Arab Minch University, Ethiopia. The experiment was carried out in a rectangular flume that is able to measure 12 m in length, 0.31 m in width, and 0.45 m in depth (Fig. 1). The flume's positive bed slope was fixed at 1:500, 1:300, and 1:200. The flume's sides are constructed with transparent Plexiglass material, while its bottom is made using steel plates. Water is pumped into the flume from an underground reservoir tank with a total capacity of 25 m^3^ by two centrifugal pumps with a discharge capacity of 50L/s. and ultimately returns to the tank at the channel end. The water is also supplied to the main experimental flume from an elevated reservoir by a gravity system.

#### 2.1.1 Sieve analysis and sampling

**Fig. 2:**
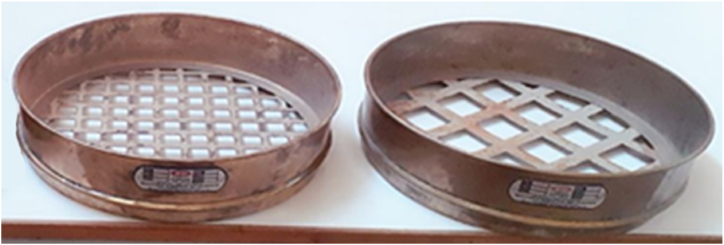
The size of sieves at 20 mm and 30 mm, respectively.

A rough bed condition with gravel bed particle sizes of 20 mm and 30 mm is prepared, followed by smooth bed conditions with lined concrete as bed material (Fig. 2).

#### 2.1.2 Depth measurement and digital balance

The overall depth was measured up to 70 mm (70 mm), and the upstream water head was measured every 5 mm with hooks and point gauges. A set of flow depths of 5, 10, 15, 20, 25, 30, 35, 40, 45, 50, 55, 60, 65, 70 mm were used at the beginning of the test area. A highly sensitive digital balance was also used to measure the mass of water flowing over bed material from upstream to downstream by collecting the flowing water in containers.

### 2.2 Roughness bed material design and construction

**Fig. 3:**
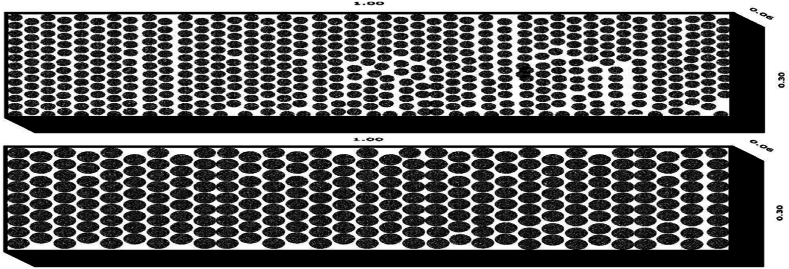
Randomly bed configuration models.

**Fig. 4:**
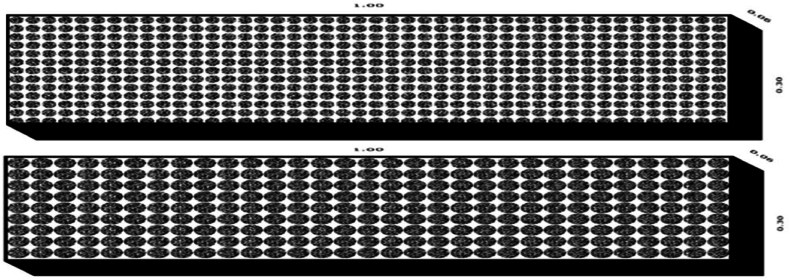
Perpendicular to flow bed configuration models.

This experimental research considered two types of grain size comprising of 20 mm and 30 mm bed materials. The first set of experiments on the random arrangement of bed material were conducted by laying a roughness plate on the flow channel and were repeated for channel slopes equal to 1:500, 1:300, and 1:200, and repeated for the second sets of experiments, perpendicular to the flow bed configuration under the same bed slope with and without weir. Those bed models were constructed from different coarse aggregates (30 mm and 20 mm) with different dimensions (0.065 m*1 m), thickness, and length including a standard rectangular weir (Fig. 3, Fig. 4).

### 2.3 Run procedure for determination of hydraulic coefficient

Water was pumped into a tank to maintain a consistent water level at the beginning of each test. The flow rate was regulated using valves on the supply pipes connected to the constant head tank. When the shutoff valves on the supply pipes were turned on and off, different flow rates and Froude numbers were observed in the channel. The water enters the channel from the supply pipes via a small basin, where a portion of the flow's energy is lost. Measurements were taken after allowing sufficient time for the flow to reach steady state conditions. The gate valve and pump switch were adjusted, with the gate valve opening controlled during the experiments. The flow depth was measured using a hook and point gauge, while the flow volume was measured using a digital scale. The time taken for the flow to travel through the experimental channel was measured using a stopwatch. Throughout the course of the experiment, the slope or gradient of the flume was maintained at a constant value. This ensured that the channel geometry and bed incline remained fixed during the data collection process.The pump was turned off after recording time to measure the depth of flow over the bed, mass of water, and flow rate before calculating the hydraulic coefficient using Manning and discharge equations, and the preceding steps were repeated for each run based on changing bed slope, bed material grain size, and bed configuration with and without a weir. This procedure was used for each of the 1680 tests in this study for the rough gravel bed surface (20 and 30 mm grain size) under various bed configurations and channel slopes with and without weir, while keeping the bed slope constant (see Table 1).

## 3 Results and Discussion

### 3.1 Influence of bed materials on roughness coefficient

The parameters of bed roughness (n) coefficients, coefficient of discharge (Cd), and hydraulic parameter values are critical in selecting the grain size of the bed material, bed arrangement, and slope of lined artificial channels. These factors are critical for determining the suitability of various hydraulic structures ([24]; Lau and Afshar, 2013; Hari Wibowo, Suripin, and Isdiyana, 2015; Ibrahim and Abdel-Mageed, 2014; [25]). The hydraulic coefficient was calculated using experimental data. Table 2 presents the various measurements and outcomes of Manning's roughness coefficient gathered during the experiment conducted on bed materials with grain size distributions of 20 mm and 30 mm. The experiment considered a bed slope of 1:500, and the bed configurations were randomly oriented and perpendicular to the flow. Additionally, the experiment was conducted both with and without a weir.

**Table 1:** Experiments conditions.

Randomly bed configurations			Perpendicular to flow bed configurations	
Slope	Test	Bed Surfaces	Test	Bed Surfaces
1:200	1	20 mm grain size	3	20 mm grain size
	2	30 mm grain size	4	30 mm grain size
1:300	5	20 mm grain size	7	20 mm grain size
	6	30 mm grain size	8	30 mm grain size
1:500	9	20 mm grain size	11	20 mm grain size
	10	30 mm grain size	12	30 mm grain size

**Table 2:** Roughness coefficient and discharge values for various bed surfaces at a bed slope of 1:500.

Randomly arranged bed configuration								
Bed Surface	With Weir				Without Weir			
Grain size (mm)	Range, n		Average, n	Q (m^3^/s)	Range, n		Average, n	Q (m^3^/s)
	From	To			From	To		
20	0.011	0.022	0.021	0.0034	0.01	0.022	0.019	0.0037
30	0.013	0.027	0.022	0.0029	0.011	0.026	0.021	0.0033
**Perpendicularly arranged bed configuration**								
20	0.015	0.033	0.027	0.0024	0.014	0.028	0.024	0.0029
30	0.025	0.038	0.033	0.0021	0.021	0.029	0.026	0.0025

Note: n = Manning roughness coefficient and Q = discharge (m^3^/s).

Table 2 presents roughness coefficients (n) and discharge values (Q) for randomly arranged and perpendicularly arranged bed configurations at a bed slope of 1:500. The table includes data for different grain sizes (20 mm and 30 mm) and whether a weir is present or not. The roughness coefficients and discharge values vary within specific ranges for each scenario. According to Thappeta et al. [3], there is a positive correlation between the grain size of bed materials and the Manning roughness coefficient in both randomly and systematically arranged bed configurations. As the grain size increases, the roughness of the bed surface also increases. This implies that larger grains result in a rougher bed surface, which in turn affects the flow characteristics and increases the resistance to flow. As a consequence, when working with bed materials of larger grain sizes, it is important to consider the corresponding increase in the Manning roughness coefficient for accurate hydraulic modelling and analysis. According to multiple studies ([[26], [27], [28], [29]]; Salah Abd Elmoaty & T. A., 2020), there is a consistent finding that the discharge value decreases in both beds as the aggregate size of the gravel bed increases. These studies show that larger aggregate sizes result in a rougher bed surface, leading to increased friction and resistance to flow. As a result, the flow velocities decrease, resulting in a reduction in the discharge value. It is important to consider this relationship between grain size and discharge when studying or designing hydraulic systems involving gravel bed materials. The transition from a randomly arranged bed configuration to a perpendicular to flow bed configuration, both with and without a weir, resulted in an increase in the average Manning roughness coefficient and a reduction in the discharge value ([29]; Salah Abd Elmoaty & T. A, 2020). This finding is consistent across the different scenarios examined in the study. Simultaneously, a decrease in discharge value indicates a reduction in flow efficiency. The perpendicular bed arrangement, which increases roughness and blockage, reduces the flow's capacity to maintain greater velocities and volumes. This decrease in flow could have an influence on the system's overall hydraulic performance, including flood management, water conveyance, and sediment transport ([2]; Roushangar & Shahnazi, 2021). When compared to a 30 mm grain size gravel bed under different bed configurations (with and without a weir), the roughness coefficient is highest in the perpendicular to flow bed configuration, independent of the presence of a weir. This indicates that the perpendicular bed arrangement, with or without a weir, generates more impediments and imperfections, resulting in increased friction and roughness. Bed arrangement, weir presence, and grain size all have a major influence on discharge values and Manning roughness coefficients in hydraulic systems (Aqeel Al-Adili,Ali Sadiq And Fadia Faroq, 2015 [2]; Study et al., 2015, 2017; [3]). For a 20-mm grain size, a perpendicular flow bed arrangement produces larger discharges and Manning roughness coefficients than a randomly distributed bed configuration. The presence of a weir has an additional effect on these numbers as well. These findings indicate the need to take these aspects into consideration when contracting hydraulic systems.

The roughness coefficient and discharge values for various bed surfaces are presented in Table 3 for a bed slope of 1:300. The data has been divided into two categories: bed surfaces with and without weirs. Additionally, the values are categorized according to the grain size of the bed surface and whether the bed structure is random or perpendicular to flow. The results of the research indicate that there are significant fluctuations in the average values of Manning's roughness coefficient and discharge for bed slopes of 1:500 and 1:300 for each test, especially when comparing bed surfaces with and without weirs. The presence or absence of weirs has a considerable effect on Manning's roughness coefficient and discharge averages on bed surfaces at slopes of 1:500 and 1:300. Weirs generate variations in flow properties such as velocity, turbulence, and resistance, resulting in various roughness coefficients and discharge values ([2]; Study et al., 2015; [3]). Bed surfaces without weirs showed significant variations in these values, demonstrating the effect of flow patterns and hydraulic conditions. Addressing these variances becomes essential to conducting accurate hydraulic analysis and design in open channels. These findings illustrate the need to consider and account for bed slope effects when analyzing and planning open channels in order to appropriately measure flow rates and hydraulic efficiency. In both bed layouts, with and without a weir, the roughness coefficient (n) increases with increasing particle size, nevertheless the discharge value decreases with greater grain sizes on the moderate bed slope [[30], [31], [32]]. The configuration of the channel bed influences how water flows through it ([[33], [34], [35]]; Sanz-Ramos et al., 2021; [36]). When the bed is perpendicular to the flow direction, it introduces new impediments and disturbances that interfere with the smooth flow of water. This increased resistance results in a greater Manning roughness coefficient, which measures the friction between the water and the channel bed. As a result, a greater roughness coefficient indicates that more energy is necessary for the water to overcome resistance and continue to flow [3,37]. As a result, a perpendicular bed configuration could result in lower discharge values, showing a slower rate of water flow through the channel. The Manning roughness coefficient for a 30 mm particle was 0.033 with a weir and 0.031 without a weir in a perpendicular flow bed design. Under a random bed configuration and moderate bed slope, the roughness coefficient was 0.026 with a weir and 0.025 without a weir. The Manning roughness coefficient quantifies the roughness or resistance to flow within a channel (Roushangar & Shahnazi, 2021). In the present research, the coefficient values show that the presence of a weir and a perpendicular flow bed configuration result in greater roughness coefficients than a random bed design with a moderate slope. This indicates that the flow encounters increased resistance and friction in the presence of a weir and a perpendicular bed configuration[35].The significant variations in roughness coefficients with and without weirs in both bed layouts indicate that the weir has significate effect on the channel's overall roughness ([38]; Salah Abd Elmoaty & T. A., 2020; [25,39]). The flow rate has an inverse proportional relationship with the Manning's roughness coefficient. As the roughness coefficient increases, the discharge decreases, and vice versa [14,40]. When the channel bed material has a significantly higher roughness coefficient, the discharge value is consistently reduced [41]. In both weir and no-weir scenarios, higher Manning's roughness coefficients are related with lower discharges. Increased roughness coefficients cause slower flow velocities and higher energy losses, resulting in lower discharge values.This relationship holds true for various fixed bed materials (Aqeel Al-Adill et al., 2015; [34,36,42]).

**Table 3:** Roughness coefficient discharge values for various Bed Surfaces at a 1:300 bed slope.

Randomly bed form or bed configuration								
Bed Surface	With Weir				Without Weir			
Grain size (mm)	Range, n		Average, n	Q (m^3^/s)	Range, n		Average, n	Q (m^3^/s)
	From	To			From	To		
20	0.012	0.027	0.022	0.0044	0.011	0.028	0.021	0.0039
30	0.014	0.028	0.026	0.0034	0.013	0.032	0.025	0.0036
**Perpendicular to Flow Bed Configuration or Bed forms**								
20	0.016	0.039	0.028	0.0028	0.015	0.033	0.027	0.0034
30	0.026	0.040	0.033	0.0024	0.022	0.035	0.031	0.0027

Note: n = Manning roughness coefficient and Q = discharge (m^3^/s).

Table 4 illustrates Manning's roughness coefficients and discharge quantities for various combinations of random bed and perpendicular to flow bed configurations tested in the trials, both with and without a weir, at a bed slope of 1:200. The results indicate that Manning's roughness coefficient and discharge values vary considerably between tests in both random bed and perpendicular to flow bed layouts. The observed changes in Manning's roughness coefficients and discharge values indicate the effect of bed configuration on flow characteristics([33,43]; Salah Abd Elmoaty & T. A, 2020). Random and perpendicular bed layouts present variable hydraulic conditions, resulting in differences in roughness coefficients and discharge quantities. Flow resistance, turbulence, and channel form all contribute to these variances([33]; Sanz-Ramos et al., 2021). Understanding these variations is essential for accurate hydraulic analysis and channel design in real-world situations[44,45].

**Table 4:** Roughness coefficient discharge values for various Bed Surfaces at a 1:200 bed slope.

Randomly bed form or bed configuration								
Bed Surface	With Weir				Without Weir			
Grain size (mm)	Range, n		Average, n	Q (m^3^/s)	Range, n		Average, n	Q (m^3^/s)
	From	To			From	To		
20	0.013	0.031	0.023	0.0043	0.012	0.029	0.022	0.0052
30	0.015	0.032	0.028	0.0036	0.014	0.031	0.027	0.0042
**Perpendicular to Flow Bed Configuration or Bed forms**								
20	0.021	0.045	0.036	0.0032	0.016	0.034	0.029	0.0038
30	0.036	0.049	0.045	0.0028	0.023	0.038	0.033	0.0032

Note: n = Manning roughness coefficient and Q = discharge (m^3^/s).

Under the bed slope of 1:200, the average values of the Manning roughness coefficient and discharge differ from those under the bed slopes of 1:500 and 1:300. The variation in average Manning roughness coefficient and discharge values between different bed slopes indicates the channel bed slope has a substantial influence on flow properties[42,46]. These findings illustrate the necessity of considering bed slope into account when evaluating and constructing channels, since it has an essential effect on the overall flow behaviors and performance of the system. Manning's roughness coefficient and discharge values do not remain constant across the channel when bed materials, layouts, and slopes change, whether with or without a weir. The variation in Manning's roughness coefficient and discharge values demonstrates the importance of bed materials, layouts, and slopes on flow characteristics. These variables influence variations in flow resistance, turbulence, and channel shape, resulting in changes in roughness and discharge across the channel. It stresses the need to take these elements into account while conducting hydraulic analysis and channel design in order to properly forecast and optimize flow behaviors [45,47]. When the bed configuration was adjusted from random to perpendicular to the flow bed pattern with and without weir, both the roughness coefficient and discharge dropped [26]. The coefficient of roughness was increased when the channel bed contains high roughness material [18]. Experiments conducted on a steeper channel slope (1:200) investigated the relationship between the bed roughness and the roughness coefficient. Two grain sizes (20 mm and 30 mm) were tested in randomly selected bed layouts with a weir. The findings revealed that bed roughness affected Manning's coefficient and flow rate in both bed layouts, with and without a weir. The 30 mm grain size gravel bed has the greatest roughness coefficient when compared to the 20 mm gravel bed. This shows that larger grain sizes generate more flow resistance and turbulence[25,48]. Understanding these facts is critical for doing proper hydraulic analysis and channel design on steep slopes. The coefficient of roughness was larger when the channel bed had high-roughness material [49,50]. Each type of bed material has its own roughness coefficient, which increases as the bed surface becomes rougher (Boorsema et al., 2017).

### 3.2 Effect of bed slope on the roughness coefficient

**Fig. 5:**
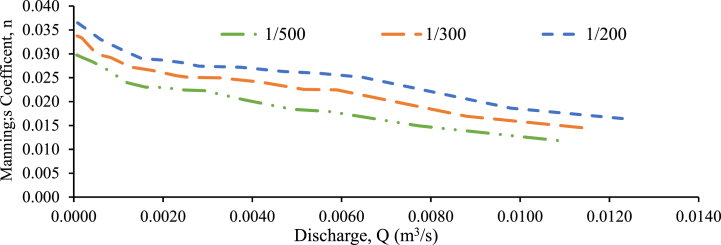
Manning's coefficient versus discharge for 30 mm grain size random bed configuration with rectangular weir.

**Fig. 6:**
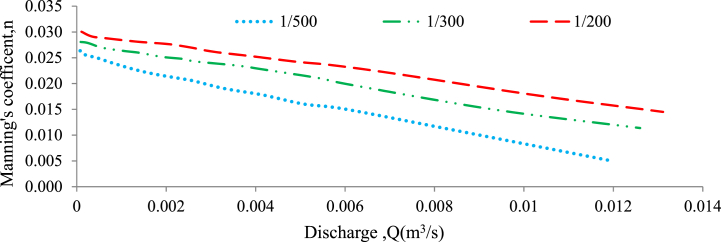
Manning's coefficient versus discharge for 20 mm grain size random bed configuration with rectangular weir.

In open channel flow, the bed slope influences the roughness coefficient significantly. As the bed slope increases, so does the roughness coefficient[51]. This is because a higher slope causes more turbulence and flow disturbances, which increases flow resistance. In the present research, the effect of channel bed slope on roughness coefficient has been examined for 30 mm and 20 mm grain size gravel beds with and without a weir, under random bed configuration and perpendicular to flow bed configuration. Fig. 5, Fig. 6 show the effect of bed slope on the roughness coefficient of a 30 mm grain size gravel surface and 20 mm with a rectangular weir under randomly chosen bed configurations, respectively, with different bed slopes (1:500, 1:300, and 1:200.These figures particularly examine the relationship between Manning's roughness, discharge, and various bed slopes (1:500, 1:300, and 1:200) in a randomly chosen bed configuration with weir.

The value of Manning's coefficient (n) reduces from a steeper slope to a flatter slope (Fig. 5, Fig. 6), which is consistent with Manning's idea (Ke et al., 2018; Ahmed et al., 2017). Manning's theory argues that as the bed slope becomes less steep (flatter), the flow faces less resistance from the channel bed, resulting in a lower Manning coefficient. This trend is supported by the decreasing Manning's coefficient values observed in Fig. 5, Fig. 6, which occur as the bed slope becomes less steep [43]. In a randomly chosen bed layout, channels with a rectangular weir slope of 1:200 have a greater roughness coefficient than channels with weir slopes of 1:300 or 1:500. The results show that a steeper slope of 1:200 in channels with a rectangular weir result in a greater roughness coefficient (Aberle & Smart, 2003; [52]). This means that flow faces higher resistance and turbulence in channels with a 1:200 slope, resulting in a rougher channel bed[34]. Channels with weir slopes of 1:300 and 1:500 have lower roughness coefficients, indicating smoother flow conditions because of the less steep bed slopes [46,53]. The effect of bed slope on the Manning coefficient demonstrates that as the bed slope flattens (1:500), the roughness value decreases. The observed drop in the Manning coefficient as the bed slope increases indicates that channels with flatter slopes have smoother flow conditions and reduced resistance to flow. A flatter bed slope reduces turbulence and flow disturbances, which leads to a lower roughness evaluation ([54]; Sanz-Ramos et al., 2021).

The observed pattern in the numbers shows that as the bed slopes steeper, flow velocity rises, resulting in increasing discharge rates. This is acceptable with the principles of open channel flow, which state that a steeper slope provides more driving force for the flow, resulting in higher velocities and flow rates[51,52]. As the slope of open channel flow rises, so does the flow velocity. As velocity increases, the Manning roughness coefficient, which indicates flow resistance, rises. Furthermore, faster velocity has the potential to result in a greater discharge rate. Generally, as the bed slope increases, the roughness coefficient also increases (Merry,2017; Lau,2018). This is due to the changes in flow characteristics that occur as the slope becomes steeper. When the bed slope increases, the flow velocity also increases. The higher velocity results in greater shear stress on the bed surface, leading to more intense bed erosion and increased roughness ([43]; Salah Abd Elmoaty & T. A, 2020; [55]). As a result, the roughness coefficient tends to increase with steeper bed slopes. Manning's roughness coefficient is inversely proportional to discharge, slopes, and bed materials. The manning coefficient is increased as the channel slope changes from flat to steeper([56]; Al-taee, 2011; Hari Wibowo, Suripin & Isdiyana, 2015; Salah Abd Elmoaty & T. A., 2020). At a given channel slope, a channel with a perpendicular flow bed configuration has a higher roughness coefficient value than randomly selected bed configurations without a weir. This indicates that the resistance to flow in the channel with a perpendicular flow bed layout is greater than that of randomly selected bed configurations with and without a weir. On the other hand, randomly chosen bed configurations with and without a weir have a lower roughness coefficient value, suggesting that the resistance to flow is lower in such cases. Manning's roughness coefficient increases when the grain size of the bed material and the channel slope are increased at the same time [52]. As a result, the flow gets increasingly blocked and inefficient [51]. These parameters are of major significance for hydraulic engineering applications such as river management, flood flow prediction, and channel capacity determination ([38,44]; Salah Abd Elmoaty & T. A, 2020; [25]). Engineers and researchers must consider these aspects when modelling and predicting water flow behaviour in channels with different bed material sizes and slopes [11,12,57].The effect of channel slope on roughness decreases gradually from steeper to flatter slope and bed material size increased manning's roughness coefficient value also increased [9,12,25,25].‬‬‬‬‬‬‬‬‬‬‬‬‬‬‬‬‬‬‬‬‬‬‬‬‬‬‬‬‬‬‬‬‬‬‬‬‬‬‬‬‬‬‬‬‬‬‬‬‬‬‬‬‬‬‬‬‬‬‬‬‬‬‬‬‬‬‬‬‬‬‬‬‬‬‬‬‬‬‬‬‬‬‬‬‬‬‬‬‬‬‬‬‬‬‬‬‬‬‬‬‬‬‬‬‬‬‬‬‬‬‬‬‬‬‬‬‬‬‬‬‬‬‬‬‬‬‬‬‬‬‬‬‬‬‬‬‬‬‬‬‬‬‬‬‬‬‬‬‬‬‬‬‬‬‬‬‬‬‬‬‬‬‬‬‬‬‬‬‬‬‬‬‬‬‬‬‬‬‬‬‬‬‬‬‬‬‬‬‬‬‬‬‬‬‬‬‬‬‬‬‬‬‬‬‬‬‬‬‬‬‬‬‬‬‬‬‬‬‬‬‬‬‬‬‬‬‬‬‬‬‬‬‬‬‬‬‬‬‬‬‬‬‬‬‬‬‬‬‬‬‬‬‬‬‬‬‬‬‬‬‬‬‬‬‬‬‬‬‬‬‬‬‬‬‬‬‬‬‬‬‬‬‬‬‬‬‬‬‬‬‬‬‬‬‬‬‬‬‬‬‬‬‬‬‬‬‬‬‬‬‬‬‬‬‬‬‬‬‬‬‬‬‬‬‬‬‬‬‬‬‬‬‬‬‬‬‬‬‬‬‬‬‬‬‬‬‬‬‬‬‬‬‬‬‬‬‬‬‬‬‬‬‬‬‬‬‬‬‬‬‬‬‬‬‬‬‬‬‬‬‬‬‬‬‬‬‬‬‬‬‬‬‬‬‬‬‬‬‬‬‬‬‬‬‬‬‬‬‬‬‬‬‬‬‬‬‬‬‬‬‬‬‬‬‬‬‬‬‬‬‬‬‬‬‬‬‬‬‬‬‬‬‬‬‬‬‬‬‬‬‬‬‬‬‬‬‬‬‬‬‬‬‬‬‬‬‬‬‬‬‬‬‬‬‬‬‬‬‬‬‬‬‬‬‬‬‬‬‬‬‬‬‬‬‬‬‬‬‬‬‬‬‬‬‬‬‬‬‬‬‬‬‬‬‬‬‬‬‬‬‬‬‬‬‬‬‬‬‬‬‬‬‬‬‬‬‬‬‬‬‬‬‬‬‬‬‬‬‬‬‬‬‬‬‬‬‬‬‬‬‬‬‬‬‬‬‬‬‬‬‬‬‬‬‬‬‬‬‬‬‬‬‬‬‬‬‬‬‬‬‬‬‬‬‬‬‬‬‬‬‬‬‬‬‬‬‬‬‬‬‬‬‬‬‬‬‬‬‬‬‬‬‬‬‬‬‬‬‬‬‬‬‬‬‬‬‬‬‬‬‬‬‬‬‬‬‬‬‬‬‬‬‬‬‬‬‬‬‬‬‬‬‬‬‬‬‬‬‬‬‬‬‬‬‬‬‬‬‬‬‬‬‬‬‬‬‬‬‬‬‬‬‬‬‬‬‬‬‬‬‬‬‬‬‬‬‬‬‬‬‬‬‬‬‬‬‬‬‬‬‬‬‬‬‬‬‬‬‬‬‬‬‬‬‬‬‬‬‬‬‬‬‬‬‬‬‬‬‬‬‬‬‬‬‬‬‬‬‬‬‬‬‬‬‬‬‬‬‬‬‬‬‬‬‬‬‬‬‬‬‬‬‬‬‬‬‬‬‬‬‬‬‬‬‬‬‬‬‬‬‬‬‬‬‬‬‬‬‬‬‬‬‬‬‬‬‬‬‬‬‬‬‬‬‬‬‬‬‬‬‬‬‬‬‬‬‬‬‬‬‬‬‬‬‬‬‬‬‬‬‬‬‬‬‬‬‬‬‬‬‬‬‬‬‬‬‬‬‬‬‬‬‬‬‬‬‬‬‬‬‬‬‬‬‬‬‬‬‬‬‬‬‬‬‬‬‬‬‬‬‬‬‬‬‬‬‬‬‬‬‬‬‬‬‬‬‬‬‬‬‬‬‬‬‬‬‬‬‬‬‬‬‬‬‬‬‬‬‬‬‬‬‬‬‬‬‬‬‬‬‬‬‬‬‬‬‬‬‬‬‬‬‬‬‬‬‬‬‬‬‬‬‬‬‬‬‬‬‬‬‬‬‬‬‬‬‬‬‬‬‬‬‬‬‬‬‬‬‬‬‬‬‬‬‬‬‬‬‬‬‬‬‬‬‬‬‬‬‬‬‬‬‬‬‬‬‬‬‬‬‬‬‬‬‬‬‬‬‬‬‬‬‬‬‬‬‬‬‬‬‬‬‬‬‬‬‬‬‬‬‬‬‬‬‬‬‬‬‬‬‬‬‬‬‬‬‬‬‬‬‬‬‬‬‬‬‬‬‬‬‬‬‬‬‬‬‬‬‬‬‬‬‬‬‬‬‬‬‬‬‬‬‬‬‬‬‬‬‬‬‬‬‬‬‬‬‬‬‬‬‬‬‬‬‬‬‬‬‬‬‬‬‬‬‬‬‬‬‬‬‬‬‬‬‬‬‬‬‬‬‬‬‬‬‬‬‬‬‬‬‬‬‬‬‬‬‬‬‬‬‬‬‬‬‬‬‬‬‬‬‬‬‬‬‬‬‬‬‬‬‬‬‬‬‬‬‬‬‬‬‬‬‬‬‬‬‬‬‬‬‬‬‬‬‬‬‬‬‬‬‬‬‬‬‬‬‬‬‬‬‬‬‬‬‬‬‬‬‬‬‬‬‬‬‬‬‬‬‬‬‬‬‬‬‬‬‬‬‬‬‬‬‬‬‬‬‬‬‬‬‬‬‬‬‬‬‬‬‬‬‬‬‬‬‬‬‬‬‬‬‬‬‬‬‬‬‬‬‬‬‬‬‬‬‬‬‬‬‬‬‬‬‬‬‬‬‬‬‬‬‬‬‬‬‬‬‬‬‬‬‬‬‬‬‬‬‬‬‬‬‬‬‬‬‬‬‬‬‬‬‬‬‬‬‬‬‬‬‬‬‬‬‬‬‬‬‬‬‬‬‬‬‬‬‬‬‬‬‬‬‬‬‬‬‬‬‬‬‬‬‬‬‬‬‬‬‬‬‬‬‬‬‬‬‬‬‬‬‬‬‬‬‬‬‬‬‬‬‬‬‬‬‬‬‬‬‬‬‬‬‬‬‬‬‬‬‬‬‬‬‬‬‬‬‬‬‬‬‬‬‬‬‬‬‬‬‬‬‬‬‬‬‬‬‬‬‬‬‬‬‬‬‬‬‬‬‬‬‬‬‬‬‬‬‬‬‬‬‬‬‬‬‬‬‬‬‬‬‬‬‬‬‬‬‬‬‬‬‬‬‬‬‬‬‬‬‬‬‬‬‬‬‬‬‬‬‬‬‬‬‬‬‬‬‬‬‬‬‬‬‬‬‬‬‬‬‬‬‬‬‬‬‬‬‬‬‬‬‬‬‬‬‬‬‬‬‬‬‬‬‬‬‬‬‬‬‬‬‬‬‬‬‬‬‬‬‬‬‬‬‬‬‬‬‬‬‬‬‬‬‬‬‬‬‬‬‬‬‬‬‬‬‬‬‬‬‬‬‬‬‬‬‬‬‬‬‬‬‬‬‬‬‬‬‬‬‬‬‬‬‬‬‬‬‬‬‬‬‬‬‬‬‬‬‬‬‬‬‬‬‬‬‬‬‬‬‬‬‬‬‬‬‬‬‬‬‬‬‬‬‬‬‬‬‬‬‬‬‬‬‬‬‬‬‬‬‬‬‬‬‬‬‬‬‬‬‬‬‬‬‬‬‬‬‬‬‬‬‬‬‬‬‬‬‬‬‬‬‬‬‬‬‬‬‬‬‬‬‬‬‬‬‬‬‬‬‬‬‬‬‬‬‬‬‬‬‬‬‬‬‬‬‬‬‬‬‬‬‬‬‬‬‬‬‬‬‬‬‬‬‬‬‬‬‬‬‬‬‬‬‬‬‬‬‬‬‬‬‬‬‬‬‬‬‬‬‬‬‬‬‬‬‬‬‬‬‬‬‬‬‬‬‬‬‬‬‬‬‬‬‬‬‬‬‬‬‬‬‬‬‬‬‬‬‬‬‬‬‬‬‬‬‬‬‬‬‬‬‬‬‬‬‬‬‬‬‬‬‬‬‬‬‬‬‬‬‬‬‬‬‬‬‬‬‬‬‬‬‬‬‬‬‬‬‬‬‬‬‬‬‬‬‬‬‬‬‬‬‬‬‬‬‬‬‬‬‬‬‬‬‬‬‬‬‬‬‬‬‬‬‬‬‬‬‬‬‬‬‬‬‬‬‬‬‬‬‬‬‬‬‬‬‬‬‬‬‬‬‬‬‬‬‬‬‬‬‬‬‬‬‬‬‬‬‬‬‬‬‬‬‬‬‬‬‬‬‬‬‬‬‬‬‬‬‬‬‬‬‬‬‬‬‬‬‬‬‬‬‬‬‬‬‬‬‬‬‬‬‬‬‬‬‬‬‬‬‬‬‬‬‬‬‬‬‬‬‬‬‬‬‬‬‬‬‬‬‬‬‬‬‬‬‬‬‬‬‬‬‬‬‬‬‬‬‬‬‬‬‬‬‬‬‬‬‬‬‬‬‬‬‬‬‬‬‬‬‬‬‬‬‬‬‬‬‬‬‬‬‬‬‬‬‬‬‬‬‬‬‬‬‬‬‬‬‬‬‬‬‬‬‬‬‬‬‬‬‬‬‬‬‬‬‬‬‬‬‬‬‬‬‬‬‬‬‬‬‬‬‬‬‬‬‬‬‬‬‬‬‬‬‬‬‬‬‬‬‬‬‬‬‬‬‬‬‬‬‬‬‬‬‬‬‬‬‬‬‬‬‬‬‬‬‬‬‬‬‬‬‬‬‬‬‬‬‬‬‬‬‬‬‬‬‬‬‬‬‬‬‬‬‬‬‬‬‬‬‬‬‬‬‬‬‬‬‬‬‬‬‬‬‬‬‬‬‬‬‬‬‬‬‬‬‬‬‬‬‬‬‬‬‬‬‬‬‬‬‬‬‬‬‬‬‬‬‬‬‬‬‬‬‬‬‬‬‬‬‬‬‬‬‬‬‬‬‬‬‬‬‬‬‬‬‬‬‬‬‬‬‬‬‬‬‬‬‬‬‬‬‬‬‬‬‬‬‬‬‬‬‬‬‬‬‬‬‬‬‬‬‬‬‬‬‬‬‬‬‬‬‬‬‬‬‬‬‬‬‬‬‬‬‬‬‬‬‬‬‬‬‬‬‬‬‬‬‬‬‬‬‬‬‬‬‬‬‬‬‬‬‬‬‬‬‬‬‬‬‬‬‬‬‬‬‬‬‬‬‬‬‬‬‬‬‬‬‬‬‬‬‬‬‬‬‬‬‬‬‬‬‬‬‬‬‬‬‬‬‬‬‬‬‬‬‬‬‬‬‬‬‬‬‬‬‬‬‬‬‬‬‬‬‬‬‬‬‬‬‬‬‬‬‬‬‬‬‬‬‬‬‬‬‬‬‬‬‬‬‬‬‬‬‬‬‬‬‬‬‬‬‬‬‬‬‬‬‬‬‬‬‬‬‬‬‬‬‬‬‬‬‬‬‬‬‬‬‬‬‬‬‬‬‬‬‬‬‬‬‬‬‬‬‬‬‬‬‬‬‬‬‬‬‬‬‬‬‬‬‬‬‬‬‬‬‬‬‬‬‬‬‬‬‬‬‬‬‬‬‬‬‬‬‬‬‬‬‬‬‬‬‬‬‬‬‬‬‬‬‬‬‬‬‬‬‬‬‬‬‬‬‬‬‬‬‬‬‬‬‬‬‬‬‬‬‬‬‬‬‬‬‬‬‬‬‬‬‬‬‬‬‬‬‬‬‬‬‬‬‬‬‬‬‬‬‬‬‬‬‬‬‬‬‬‬‬‬‬‬‬‬‬‬‬‬‬‬‬‬‬‬‬‬‬‬‬‬‬‬‬‬‬‬‬‬‬‬‬‬‬‬‬‬‬‬‬‬‬‬‬‬‬‬‬‬‬‬‬‬‬‬‬‬‬‬‬‬‬‬‬‬‬‬‬‬‬‬‬‬‬‬‬‬‬‬‬‬‬‬‬‬‬‬‬‬‬‬‬‬‬‬‬‬‬‬‬‬‬‬‬‬‬‬‬‬‬‬‬‬‬‬‬‬‬‬‬‬‬‬‬‬‬‬‬‬‬‬‬‬‬‬‬‬‬‬‬‬‬‬‬‬‬‬‬‬‬‬‬‬‬‬‬‬‬‬‬‬‬‬‬‬‬‬‬‬‬‬‬‬‬‬‬‬‬‬‬‬‬‬‬‬‬‬‬‬‬‬‬‬‬‬‬‬‬‬‬‬‬‬‬‬‬‬‬‬‬‬‬‬‬‬‬‬‬‬‬‬‬‬‬‬‬‬‬‬‬‬‬‬‬‬‬‬‬‬‬‬‬‬‬‬‬‬‬‬‬‬‬‬‬‬‬‬‬‬‬‬‬‬‬‬‬‬‬‬‬‬‬‬‬‬‬‬‬‬‬‬‬‬‬‬‬‬‬‬‬‬‬‬‬‬‬‬‬‬‬‬‬‬‬‬‬‬‬‬‬‬‬‬‬‬‬‬‬‬‬‬‬‬‬‬‬‬‬‬‬‬‬‬‬‬‬‬‬‬‬‬‬‬‬‬‬‬‬‬‬‬‬‬‬‬‬‬‬‬‬‬‬‬‬‬‬‬‬‬‬‬‬‬‬‬‬‬‬‬‬‬‬‬‬‬‬‬‬‬‬‬‬‬‬‬‬‬‬‬‬‬‬‬‬‬‬‬‬‬‬‬‬‬‬‬‬‬‬‬‬‬‬‬‬‬‬‬‬‬‬‬‬‬‬‬‬‬‬‬‬‬‬‬‬‬‬‬‬‬‬‬‬‬‬‬‬‬‬‬‬‬‬‬‬‬‬‬‬‬‬‬‬‬‬‬‬‬‬‬‬‬‬‬‬‬‬‬‬‬‬‬‬‬‬‬‬‬‬‬‬‬‬‬‬‬‬‬‬‬‬‬‬‬‬‬‬‬‬‬‬‬‬‬‬‬‬‬‬‬‬‬‬‬‬‬‬‬‬‬‬‬‬‬‬‬‬‬‬‬‬‬‬‬‬‬‬‬‬‬‬‬‬‬‬‬‬‬‬‬‬‬‬‬‬‬‬‬‬‬‬‬‬‬‬‬‬‬‬‬‬‬‬‬‬‬‬‬‬‬‬‬‬‬‬‬‬‬‬‬‬‬‬‬‬‬‬‬‬‬‬‬‬‬‬‬‬‬‬‬‬‬‬‬‬‬‬‬‬‬‬‬‬‬‬‬‬‬‬‬‬‬‬‬‬‬‬‬‬‬‬‬‬‬‬‬‬‬‬‬‬‬‬‬‬‬‬‬‬‬‬‬‬‬‬‬‬‬‬‬‬‬‬‬‬‬

### 3.3 Effect of roughness on discharge

The effect of roughness on discharge in open channels is well understood in hydraulic engineering. This research showed that Manning's roughness coefficient (n) is a quantitative indicator of the link between bed roughness and flow discharge. Manning's roughness coefficient values are higher, indicating rougher channel conditions and lower discharge values. Table 5 summarizes flow rates depending on Manning's roughness coefficient and slopes for random and perpendicular bed configurations. It includes the coefficient (n), reciprocal (1/n), area, perimeter (P), radius (R), slope (S), and discharge (Q) for each test scenario. Tests 1, 2, 5, 6, 9, and 10 employed 20 mm and 30 mm grain size models with a random bed configuration for slopes 1:200, 1:300, and 1:500. Tests 3, 4, 7, 8, 11, and 12 utilized 20 mm and 30 mm grain size bed models with a perpendicular flow bed configuration. Test 1 (1:200) with a randomly selected bed layout and a 20 mm grain size gravel bed exhibited the highest rate of flow (0.0040 m3/s) as compared to Test 3 (1:200) with a perpendicular to flow bed configuration and the same grain size. This indicates that the random bed pattern may have lower hydraulic roughness, leading to higher flow rates ([28]; S. M. Borghei, M. R. Jalili, 1999; [25,58]). The experiments indicate that the 20 mm grain size bed model exhibits a lower roughness coefficient compared to the 30 mm grain size bed model. This lower roughness coefficient results in a reduced retarding effect on the water flow. As a consequence, higher flow rates are observed for both bed configurations (with weir and without weir) when using the 20 mm grain size bed model. The 20-mm grain size bed model has a reduced roughness coefficient, implying that there are fewer blockages or imperfections on the bed surface that prevent water from flowing smoothly. This enables for a more effective movement of water, which leads to higher flows. This effect is seen in both bed designs, demonstrating that the impact of grain size on flow rates is constant across bed.

**Table 5:** The discharge of different bed roughness for specific slopes using hydraulic parameters with weir in random bed and perpendicular to flow bed configurations.

Slope	Test	Manning's n	1/n	Area (m^2^)	P (m)	R (m)	R^2/3^	Slope	S^1/2^	Q (m^3^/s)
1:200	1	0.0252	39.69	0.01125	0.375	0.029	0.0935	0.01	0.07071	0.004
	2	0.0277	36.1	0.01125	0.375	0.029	0.0935	0.01	0.07071	0.0038
	3	0.0415	24.07	0.01125	0.375	0.029	0.0935	0.01	0.07071	0.0023
	4	0.0472	21.2	0.01125	0.375	0.029	0.0935	0.01	0.07071	0.002
1:300	5	0.0229	43.69	0.01125	0.375	0.029	0.0935	0	0.05774	0.0037
	6	0.0254	39.34	0.01125	0.375	0.029	0.0935	0	0.05774	0.0035
	7	0.0318	31.44	0.01125	0.375	0.029	0.0935	0	0.05774	0.0025
	8	0.0395	25.33	0.01125	0.375	0.029	0.0935	0	0.05774	0.0019
1:500	9	0.0199	50.36	0.01125	0.375	0.029	0.0935	0	0.04472	0.0034
	10	0.0223	44.84	0.01125	0.375	0.029	0.0935	0	0.04472	0.0032
	11	0.0272	36.78	0.01125	0.375	0.029	0.0935	0	0.04472	0.0024
	12	0.0328	30.49	0.01125	0.375	0.029	0.0935	0	0.04472	0.0018

Note: n = Manning roughness coefficient, Q = discharge (m^3^/s), R = hydraulic radius (m), P = wetted perimeter (m), A = Cross-sectional area (m^2^) and S = bed slope.

The Manning's roughness coefficient represents the energy loss due to the water friction against bed surface roughness and this roughness in bed surface increases the flow in the channel and flow velocity is strongly dependent on the resistance to flow (Mohammed, 2017; [18]). As the value of the Manning's roughness coefficient (n) increases, the velocity of the water flowing across the surface decreases. It is evident as the finishing surface of the channel became rougher, subsequently n value increased. Flow rate and roughness coefficient were influenced by bed roughness and slope and the roughness will affect the flow rate of channel in both bed configuration with weir and without weir under different type of bed slope(Aqeel Al-Adili,Ali Sadiq and Fadia Faroq, 2015; Chen et al., 2009; [2]). When the flow resistance increases as roughness coefficient increases, it produces a retarding force on the water. The channel flow rate decreases as the Manning's n increases under random bed configuration and perpendicular to the flow bed configuration (Hossieni et al., 2018; [19]). Based on the arrangement or the configuration of bed material, there are different values of roughness coefficients [59]; AL-Adili, 2013. As the roughness coefficient increased, the flow resistance also increased. This increased flow resistance created a retarding or slowing force on the water (Mohammed, 2017; [18]). Flow rate and roughness coefficient were influenced by bed roughness and bed configuration and the relation of discharge and manning roughness coefficient are inversely proportional (Hossieni et al., 2018). In general, these findings indicate that the grain size used in the bed model might have a substantial influence on the channel's roughness characteristics, which in turn affect flow rates [27,35,53,57,[60], [61], [62], [63], [64]]. Smaller grain sizes, such as 20 mm in this example, tend to provide lesser roughness and higher flow rates than bigger grain sizes.

### 3.4 Effect of Manning roughness coefficient on Froude number in open channel

In open channels, the Manning roughness coefficient influences the Froude number, which is a measure of flow regime. This research observed that as the Manning coefficient increases (showing a coarser channel bed), flow resistance rises, resulting in lower velocity. As a result, the Froude number tends to fall, indicating slower and more peaceful flow conditions [[11], [59], [65], [66], [67], [68], [69]]. This relationship is essential for evaluating flow stability and behavior in hydraulic engineering. In this experimental study, the research was conducted by testing different flow rates with varying values of flow depth and velocities. By using the continuity equation, the velocity of flow and Froude number were calculated using Equations (1) and (2) respectively [2].

(1) $$V=\frac{Q}{A}$$

(2) $$F_{r}=\frac{V}{\sqrt{\text{gy}}}$$

Whereas: V = flow velocity (m/s), A = cross-sectional area (m^2^), Q = discharge (m^3^/s), Fr = flow number, g = gravitational acceleration (m/s^2^) and y = depth of flow (m).

**Fig. 7:**
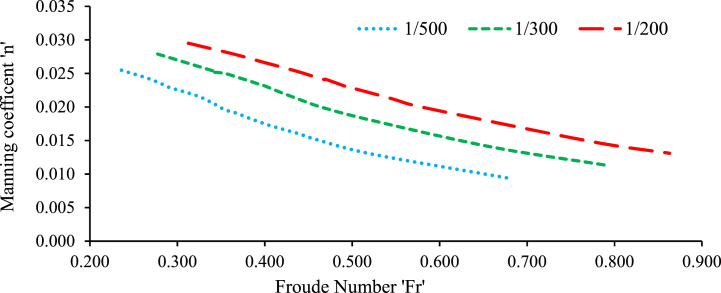
Roughness coefficient verses Froude number for a 20 mm grain size gravel bed on randomly bed configured without a weir.

**Fig. 8:**
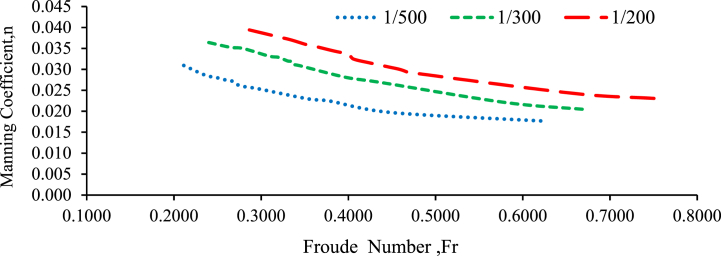
Roughness coefficient verses Froude number for a 20 mm grain size gravel bed on perpendicular to flow bed configured without a weir.

In this study, the values of the Froude number and Manning's roughness coefficient were calculated for subcritical flow conditions in the middle section of the flume. This was done for both perpendicular and randomly selected bed configurations, with and without the presence of a weir structure. The calculations were performed to characterize the flow regime and roughness properties under these varying channel bed conditions. Fig. 7, Fig. 8 were plotted to define the relationship between the Froude number and the Manning roughness coefficient without weir for both bed configurations, respectively.

In both the perpendicular to flow and random bed configurations, with and without a weir, the Froude number decreased towards the middle of the channel, the Manning's roughness coefficient was observed to increase. This finding indicates that when the flow becomes less energetic (as shown by a lower Froude number), the roughness of the channel bed has a greater influence on flow resistance[[70], [71], [72]]. In other words, at lower flow velocities, the resistance induced by irregularities and roughness elements on the bed surface becomes more pronounced, resulting in a greater Manning roughness coefficient [54]. The results indicate that as the flow depth increased the Froude number fell. On the channel side, a drop in Manning's roughness coefficient led to an increase in the Froude number[70]. Additionally, when the flow depth decreased the Froude number increased[67].

In conclusion, the study found that the 20 mm gravel bed layout has a substantial linear connection with discharge, particularly when oriented perpendicular to the flow. This is because low discharge causes high friction, resulting in a predictable polynomial fluctuation in flow [54]. Furthermore, Manning's roughness coefficient values for the perpendicular bed arrangement are often greater than those for other bed configurations, such as the randomly organized bed and the perpendicular bed with or without a weir. The highest flow velocities in an open channel are found at the centre of the channel and along the free water surface [10]. The findings suggest that when discharge values decrease in the centre of the channel, the determination factor values in subcritical flow analysis also decrease for both bed configurations. Additionally, as velocity increases at moderate Froude numbers, the effective shear stress increases as well [15]. The relationship between Froude number and friction factor provides valuable information that can be utilized in the design, implementation, and cost minimization of construction projects. Importantly, the Froude number (Fr) is inversely proportional to Manning's roughness coefficient ([45]; Sanz-Ramos et al., 2021; [36]). Froude number was computed for each depth of flow with and without weir. In summary, the findings indicate that the Froude number increased when the channel slope increased and Manning's roughness coefficient decreased, for the case without a weir and under both random and perpendicular bed configurations relative to the flow direction (Valle and Gregory, 2006).

### 3.5 The effect of particle size on the Manning's resistances coefficient

**Fig. 9:**
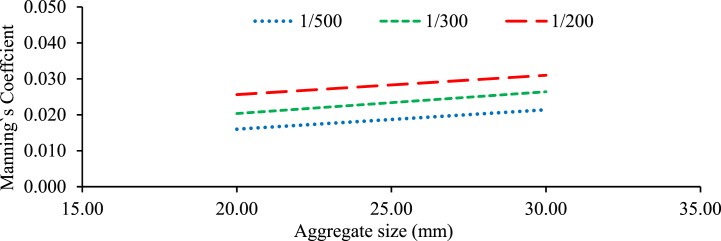
Manning's Coefficients against particle Size with different bed slope.

Fig. 9 shows the effect of bed material particle size on flow resistance (average Manning's roughness coefficient). The particle size of the bed material has significant effects on the Manning roughness coefficient. In general, as particle size expands, Manning's roughness coefficient decreases ([42,45,[46], [73], [74], [75], [76], [77]]; Salah Abd Elmoaty & T. A., 2020; [25]). The real effect of the bed on the coefficient of discharge would remain intangible as long as existing calculations are not modified to reflect the aggregate size. Resistance in a channel increase with aggregate size increases with and without a weir under different bed configuration and bed slope. As a result, Manning's roughness coefficient in a channel is proportional to grain size, and it decreases as discharge and flow depth of water increase [10]. In general, the results of this research indicate that particle size plays a role in determining the roughness characteristics of the bed, which in turn affects the flow resistance and Manning's roughness coefficient. It is important to consider the particle size when estimating Manning's roughness coefficient for a specific channel or water flow scenario [43,48].

### 3.6 Impact of Manning's roughness coefficient on Reynolds number

**Fig. 10:**
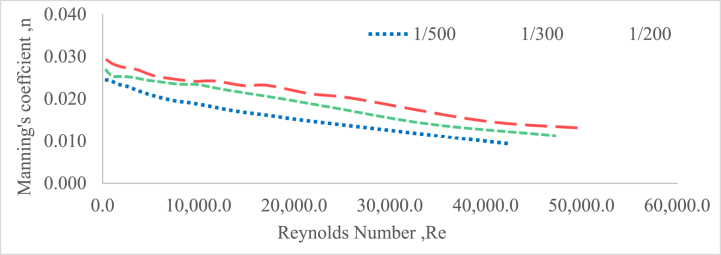
Roughness coefficient verses Reynolds number for a 20 mm gravel bed under randomly configured bed without a weir.

**Fig. 11:**
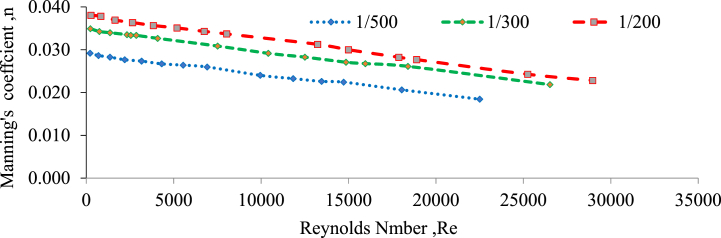
Roughness coefficient verses Reynolds number for a 20 mm gravel bed under perpendicular to flow bed configured without a weir.

According to the findings of this research, it was observed that the Manning's roughness coefficient has a direct influence on the Reynolds number[[21], [54], [56], [70], [71], [78]]. The Reynolds number is a dimensionless parameter that is widely employed to understand and describe the behaviour of fluid flow. When Manning's roughness coefficient increases, Reynolds numbers decrease, indicating a shift towards laminar or smoother flow regimes. Lower Manning's roughness coefficients, on the other hand, are associated with higher Reynolds numbers, indicating a shift towards more turbulent flow regimes(Chen et al., 2009). Fig. 10, Fig. 11 indicate the relationship between roughness coefficient and Reynolds number for a 20 mm particle size gravel material in randomly selected and perpendicular to flow bed configurations without a weir, respectively. These graphs illustrated how changes in the roughness coefficient influenced the Reynolds number in these specific bed layouts. The patterns demonstrated by the figures were evaluated to determine the general trend and nature of the link between these two variables.

For each slope and bed material, the degree of submergence, Reynolds number, and flow depth increased as Manning's Roughness Coefficient decreased. This indicates that when the roughness coefficient decreased the flow conditions were more conducive to increasing submergence, higher Reynolds numbers, and greater flow depths [[70], [79], [80]].

Additionally, the higher Reynolds numbers imply a transition towards more turbulent flow regimes, which are accompanied by increased mixing and greater energy dissipation. The results indicate a significant correlation between Manning's roughness coefficient and Reynolds number (Aberle & Smart, 2003; [51,52]). As flow speed increases, the channel's roughness reduces, resulting in higher Reynolds numbers. This means that the flow becomes more efficient and less resistance to friction [81]; Kim et al., 2007. The lines in the illustrations have comparable shapes, indicating a consistent tendency across scenarios [36]. Hydraulic roughness coefficients tend to decrease as Reynolds numbers increase. This shows that when the flow changes to more turbulent regimes, flow resistance decreases and flow efficiency increases([54,71,82]; Chen et al., 2009). The results of this research have practical applications in hydraulic engineering and fluid dynamics. They emphasize the need of evaluating roughness's effect on flow behavior, as well as selecting optimal roughness coefficients for accurate flow models and system design [33,43,54]. Understanding this relationship allows engineers to optimize flow conditions, reduce energy losses, and improve the performance of fluid transportation systems.

### 3.7 Determination of the coefficient of discharge (***Cd***)

The coefficient of discharge (Cd) is determined by measuring or estimating the efficiency of fluid flow through a particular system or apparatus ([28,83]; S. M. Borghei, M. R. Jalili, 1999). This study focused on experimental approaches for determining Cd properly. These approaches involve monitoring or simulating flow rates and pressure differentials across the system under controlled conditions. Researchers computed Cd by comparing real-world flow rates to theoretically anticipated values. For a specific type of rectangular weir, the estimation of V_1_ (velocity of approach) and H_1_ (head over the crest) can be done using Equations (1), (3)), respectively. These equations are derived from experimental results and based on the measured discharge (Q), measured head (h1), and known design parameters (b, h, g)[84].

(3) $$H_{1}=h+\frac{V_{1}^{2}}{2g}$$

Whereas: H_1_ - Upstream energy head, h -Water depth above the channel bed, V_1_- Upstream flow velocity and g - gravitational acceleration (m/s^2^).

The discharge coefficient (Cd) was determined using the standard weir equation (Equation (5)). Experimental data was collected for different bed materials, slopes, and layouts with a rectangular weir, including randomized and perpendicular to flow bed configurations. The results, including the discharge coefficients and observations, are summarized in Table 6, Table 7. The discharge coefficient for a rectangular weir was calculated using the following formula [85].

(4) Q=Cd*(2g)12BH13232

From equation (4),

(5) $$Cd=\frac{3Q}{2*{\left(2g\right)}^{\frac{1}{2}}{H1}^{\frac{3}{2}}}$$

**Table 6:** Discharge Coefficient Values for Rectangular Weir under Randomly bed configuration and different bed slope.

Slope	Bed Surface	Observed Discharge Coefficient (Cd)			
	in mm	From	To	Average, Cd	Q(m^3^/s)
1:500	20	0.400	0.879	0.558	0.0034
	30	0.356	0.816	0.499	0.0032
1:300	20	0.463	0.983	0.634	0.0037
	30	0.378	0.892	0.534	0.0035
1:200	20	0.517	1.059	0.692	0.0040
	30	0.406	0.998	0.586	0.0036

**Table 7:** Discharge Coefficient Values for Rectangular Weir and perpendicular to flow bed layouts and different bed slope.

Slope	Bed Surface	Observed Discharge Coefficient (Cd)			
	in mm	From	To	Average, Cd	Q(m^3^/s)
1:500	20	0.297	0.641	0.401	0.00237
	30	0.267	0.486	0.337	0.00183
1:300	20	0.321	0.696	0.445	0.00248
	30	0.289	0.499	0.354	0.00192
1:200	20	0.356	0.736	0.478	0.00252
	30	0.316	0.517	0.381	0.00199

Whereas: Cd - discharge coefficient, H_1_ - Upstream energy head(m), h -Water depth above the channel bed (m), g - gravitational acceleration (m/s^2^), Q – discharge (m^3^/s) and B- width of the channel (m). Table 6 illustrates the discharge coefficient values for a rectangular weir with randomly selected bed designs and slopes. The discharge coefficient (Cd) measures the efficiency of fluid flow through the weir. For each bed slope (1:500, 1:300, and 1:200), the table displays the measured discharge coefficient values for two distinct bed surface conditions (20 mm and 30 mm). The results show that when the bed slope steepens (from 1:500 to 1:200), the average discharge coefficient increases. This shows that a higher slope increases flow efficiency across the weir [84,86]. The observation that as the bed surface thickness increases from 20 mm to 30 mm, the average discharge coefficient decreases almost shows that a larger bed surface has a somewhat adverse effect on flow efficiency via the rectangular weir. A larger bed surface may provide extra obstacles or roughness factors, disrupting the smooth flow of water across the weir crest [84,[86], [87], [88]]. This can cause greater frictional losses and reduced flow efficiency, resulting in a slightly lower discharge coefficient. Moreover, with randomly and perpendicular to bed layouts, the actual coefficient of discharge across the rectangular weir for a steeper slope is higher in amount than the flatter bed slope (Mohammed, 2017; [19]). The actual coefficient of discharge over the rectangular weir in randomized bed layouts is greater compared to the perpendicular flow bed configuration. While experimental discharge value obtained for 20 mm grain size bed model under 1:200 bed slope is 0.0040 under randomly bed configuration and for under 1:500 bed slope 0.0034. Thus, rectangular weir under steeper slope and small size of gravel bed has higher discharge than under flatter bed slope and higher size gravel bed material [18].

The results suggest that the actual value of the coefficient of discharge over the rectangular weir is influenced by the channel bed configuration, bed particle size, and channel bed slopes. Additionally, the discharge coefficient is inversely proportional to the grain size of the bed material (Al-khatib and Gogus, 2014; [49]). The comparison of the observed discharge coefficient values indicates that the discharge coefficient is influenced by the configuration and slope of the bed surface. Generally, as the bed slope becomes steeper, the discharge coefficient tends to decrease(Ansari et al., 2019; [89]). Additionally, the results suggest that the grain size of the bed material plays a role in determining the discharge coefficient, with smaller grain sizes associated with lower Cd values. These findings align with previous research highlighting the influence of bed configuration, particle size, and slope on the actual value of the discharge coefficient for a rectangular weir.

Table 7 illustrates the discharge coefficient values for a rectangular weir with perpendicular to flow bed layouts designs and slopes. The discharge coefficient (Cd) measures the efficiency of fluid flow through the weir. For each bed slope (1:500, 1:300, and 1:200), the table displays the measured discharge coefficient values for two distinct bed surface conditions (20 mm and 30 mm). The discharge coefficient is also found to be directly proportional to the channel slope, indicating that as the channel slope increases, the discharge coefficient increases as well (Bategeleza, Deepak et al., 2015). The discharge coefficient increases as the actual discharge rises; however, the size of the bed material reduces as both the actual discharge and the discharge coefficient rise (Hossieni et al., 2018). The discharge coefficient and the grain size of the bed material are inversely proportional. The average discharge coefficients for the randomly chosen bed design (Table 6) are bigger than for the perpendicular to flow bed configuration (Table 7) for similar bed slopes and thicknesses. It indicates that the randomly chosen bed configuration has somewhat higher flow efficiency than the perpendicular flow bed layout [84,85,90].

### 3.8 Statistical metrics

In order to assess the performance of the rectangular weir models, the researcher compared experimental and expected discharge coefficients. This evaluation used three statistical metrics: R^2^, MAPE, and RMSE, NRMSE, and MAE [89,91]. These metrics are calculated as follows:

(6) $$MAE=\frac{1}{n}\sum_{i=1}^{n}\left|x_{i}-y_{i}\right|$$

(7) $$MAPE=\frac{\frac{100}{n}\sum_{i=1}^{n}\left|x_{i}-y_{i}\right|}{x_{i}}$$

(8) $$NRMSE=\frac{RMSE}{{\bar{x}}_{i}}$$

(9) $$RMSE=\sqrt{\frac{1}{n}\;\sum_{i=1}^{n}{\left(x_{i}-y_{i}\right)}^{2}}$$

Where MAPE is the mean average percentage error, RMSE is the root mean squared error; NRMSE is the normalized root mean number of a dataset; X_i_ is the actual discharge coefficient value, and X_mean_ is the average experimental discharge coefficient value. Y_i_ is the predicted discharge coefficient from the proposed models.

Table 8 illustrates the calculated statistical metrics for the proposed weir model in randomly selected bed configurations with varying slopes and bed surface thicknesses. The R^2^ values range from 0.9800 to 0.9998, indicating an acceptable correspond to between the predicted and experimental data. The Mean Absolute Error (MAE) values are generally small, ranging from 0.002 to 0.029. This reflects that the average absolute difference between the predicted and experimental discharge coefficient values is quite small. The MAPE values vary from 0.01 to 0.178, indicating an insignificant average percentage difference. The RMSE values vary from 0.005 to 0.020, suggesting high accuracy in the model's predictions. The NRMSE values vary from 0.004 to 0.021, indicating a minor relative error compared to the experimental discharge coefficients. The R^2^ values for all combinations are extremely near to one, suggesting a good match between predicted and experimental discharge coefficient values [28]. The MAE values are often modest, indicating a small average absolute difference between predicted and experimental discharge coefficient values[28,84,89,92]. MAPE values are likewise modest, showing that the predicted and experimental discharge coefficient values deviate by a tiny proportion on average. The RMSE values are low, indicating high accuracy in the model's predictions[34,46,54]. The NRMSE values are likewise modest, indicating a minimal relative error when compared to the range of experimental discharge coefficients values [28,84,84,88]. The 1:200 slope and 20 mm bed surface consistently exceeded all other configurations in terms of MAE, MAPE, RMSE, and NRMSE, demonstrating the model's predictions with the highest accuracy, precision, and relative accuracy. The results of this research have significance for understanding and developing hydraulic structures with weirs, resulting in better water management and technical applications.

**Table 8:** Estimated statistical metrics for the proposed weir model under randomly bed configuration.

Slopes	Bed Surface (mm)	R^2^	MAE	MAPE	RMSE	NRMSE
1:500	20	0.9990	0.010	0.094	0.013	0.018
	30	0.9800	0.029	0.178	0.020	0.021
1:300	20	0.9998	0.009	0.09	0.011	0.012
	30	0.9997	0.01	0.121	0.013	0.014
1:200	20	0.9990	0.0023	0.01	0.005	0.004
	30	0.9990	0.002	0.09	0.0078	0.008

### 3.9 Variation of discharge coefficient with Froude number

**Fig. 12:**
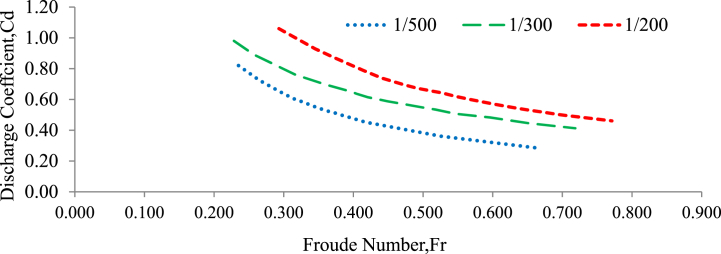
Variation of the discharge coefficient with the Froude number for a 20 mm grain size gravel bed under random bed arrangement and bed slope.

**Fig. 13:**
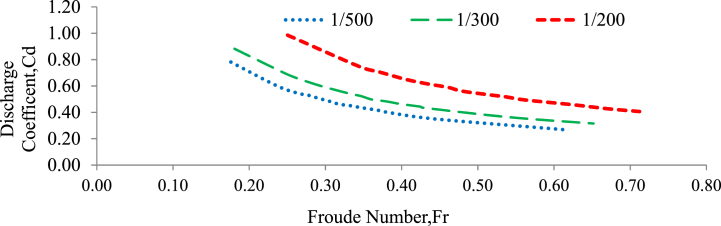
Variation of the discharge coefficient with the Froude number for a 20 mm grain size gravel bed under random bed arrangement and bed slope.

According to Konwar and Bibhash [30], the Froude number is proportional to the combined effects of inertia and gravity in a flow system. It is commonly used to differentiate between critical, subcritical, and supercritical flow regimes with F_r_ (equation (10)). The study proved the relationship between the discharge coefficient and the Froude number for gravel beds of various particle sizes. Fig. 12 depicted this relationship for gravel beds with a grain size of 20 mm, whereas Fig. 13 demonstrated the same for gravel beds with a grain size of 30 mm. The study conducted experiments in which the flow conditions were always subcritical, i.e., the Froude number (Fr) was always less than 1. In these subcritical flow circumstances, the study found a correlation between the discharge coefficient and the Froude number, which followed a power function [31]. When two variables are directly proportional, it means that as one variable increases, the other variable also increases, and vice versa. In this case, as the Froude number increased, the discharge coefficient also decreased, and as the Froude number decreased, the discharge coefficient also increased[54,70,82]. The power function relationship suggests that the changes in the Froude number have a nonlinear effect on the corresponding changes in the discharge coefficient and is described in (Equation (10)). This means that as the Froude number increases or decreases, the discharge coefficient does not change in a linear fashion, and the Froude number is determined by the following equation [67].

(10) $$Fr=\frac{V}{\sqrt{gy}}$$

Whereas; V = velocity of flow (m/s), y = depth of flow (m), g = gravity (m/s^2^) and Fr = Froude number.

In this study, it was observed that as the Froude number raised to higher values, the slope of the curve representing the relationship between the discharge coefficient (Cd) and the Froude number (Fr) for gravel beds with grain sizes of 20 mm and 30 mm (under randomly chosen bed configurations and different bed slopes) also increased gradually. This expanding slope indicates that when the Froude number increases, the correlation between Cd and Fr becomes stronger. As the Froude number increases, it appears to have a greater impact on flow behavior. This indicates that the influence of the Froude number on the discharge coefficient approaches a saturation point or a limit[54,70,71,67,82]. Beyond this threshold, additional increases in the Froude number have little effect on the discharge coefficient [28,93].

The correction value between the experimental discharge coefficient and the Froude number is R^2^ = 0.999. Generally, the R-squared value of 0.999 indicates a very strong correlation between the experimental discharge coefficient and the Froude number, suggesting that the Froude number is an excellent predictor of the discharge coefficient in the given experimental data.

### 3.10 Variation of discharge coefficient with Reynolds number

**Fig. 14:**
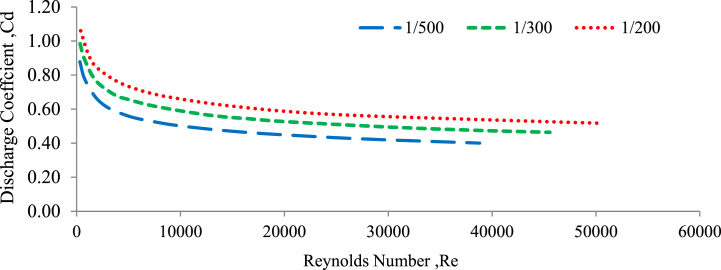
Discharge coefficient versus Reynolds number for a 20 mm randomly bed configured.

**Fig. 15:**
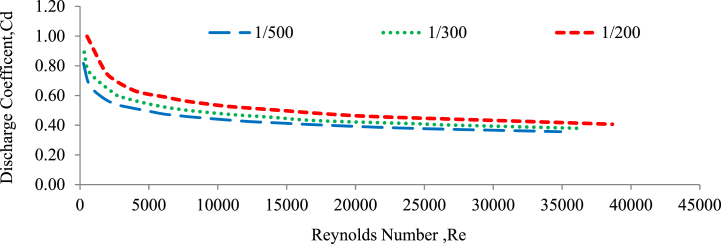
Discharge coefficient versus Reynolds number for a 30 mm randomly bed configured.

The variation of the discharge coefficient with Reynolds number is an important aspect in the study of fluid flow through various structures, including weirs. In the case of weirs, the discharge coefficient is frequently influenced by the Reynolds number. At low Reynolds numbers, the flow is usually laminar in nature, and the discharge coefficient remains relatively constant. However, as the Reynolds number increases, the flow transitions to a turbulent regime, and the discharge coefficient begins to vary accordingly (Shamshuddin & Rajput, 2023). Fig. 14, Fig. 15 illustrate the relationship between the discharge coefficient (Cd) and the Reynolds number (Re) for 20 and 30 mm under random bed configurations with three different channel slopes: 1:500, 1:300, and 1:200.

As shown, the relationship between discharge coefficient and Reynolds number is a power function, with an increase in Re resulting in a decrease in discharge coefficient ([8]; Balachandar and Ramachandran 1999; Salmasi 2022; Yamamoto and Kunugi 2012). The observations presented in Fig. 12, Fig. 13 showed that the experimental discharge coefficient (Cd) increased as the experimental Reynolds number (Re) decreased. This trend was observed for both the 20 mm and 30 mm gravel bed configurations, as well as across the three different bed slopes examined. This inverse relationship between the discharge coefficient and the Reynolds number suggests that the discharge coefficient increases as the flow regime changes from turbulent to laminar (lower Reynolds numbers). This pattern is consistent with the behaviour observed in flow systems. As the channel slope changed from flatter to steeper, under the randomly selected bed configurations, the discharge coefficient values and the Reynolds number both increased. This indicates that the coefficient of discharge and the Reynolds number are significantly influenced by the channel slope, bed configuration, and the grain size of the channel bed material (Aizhong Ye, Zheng Zhou, Jinjun You 2018; Balachandar and Ramachandran 1999; Prohaska 2008).

The relationship between the experimental discharge coefficient (Cd) and the Reynolds number (Re) follows a power function, and the average R^2^ value is 0.999, which indicates an exceptionally high degree of correlation or fit between the experimental data and the power function model. This indicates a very strong relationship between Cd and Re, with the power function model accounting for almost all of the observed variation in the experimental data. Under a 20 mm grain size gravel bed, larger values of the discharge coefficient and Reynolds number were generated compared to a 30 mm grain size grave bed, regardless of the type of channel slope.

### 3.11 Predicted discharge coefficient based on dimensional analysis technique

For any or all RW models, the discharge coefficients of rectangular notches are often dependent on geometric characteristics [[14], [94]]. The primary flow parameters are related to the important variables among numerous elements impacting the coefficient of discharge (Cd) based on the open channel flow theory. These parameters were functionally interpreted in equation (4).

(11) ƒ(Cd,H,P,g,h,ρ,fr)=0

Where; Cd= Discharge coefficient, (dimensionless), g= Gravitational acceleration ($LT^{-2}$), ρ= Mass density $\left(ML^{-3}\right)$, Fr= Froude number

Only three basic units (m = 3) are involved, illustrated as L, M, and T. There are a total of six variables, or n = 6. The following dimensionless groups emerged based on Buckingham's theorem and repeated variables [14]. According to Buckingham's theorem, (n-m) = (6-3) = 3 terms (equation (11), (12)))

(12) $$ƒ=\left(\pi_{1,}\;\pi_{2},\pi_{3}..\pi_{n-m}\right)$$

The number of variables in each term is (m+1). So (3 + 1) = 4, taking (g, H, ρ) as repeating variables, hence,

(13) $$\pi_{1}=g^{a}H^{b}\rho^{c}h$$

(14) $$\pi_{2}=g^{a}H^{b}\rho^{c}P$$

(15) $$\pi_{3}=g^{a}H^{b}\rho^{c}Cd$$

Expressing these in dimension terms for each model, we have:

(16) $$ƒ,\left(\frac{P}{H},\frac{h}{H},Cd,Fr\right)=0$$

After the simplification of the equations above, the dimensional relationship can be simplified to:

(17) $$\text{Cd}=\varphi \left(\frac{P}{H},\frac{h}{H},\text{Fr}\right)$$

Using the computer package statistical produces to perform a non-linear regression analysis of the information, the following formula is mostly used for the value of the predicted discharge coefficient [32].

(18) $$\text{Cd}=0.5767+0.0152\left(\frac{H}{P}\right)+0.01112\left(\frac{H}{h}\right)+\text{Fr}$$

**Fig. 16:**
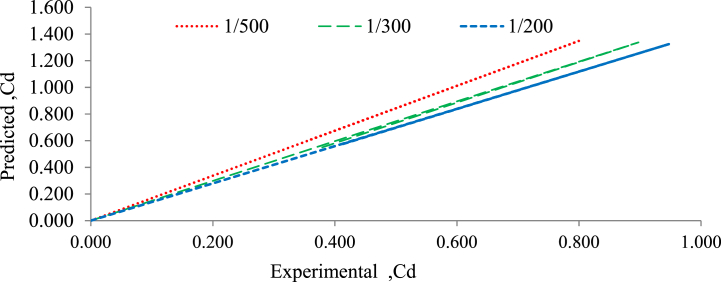
Validation of predicted and experimental coefficient of discharge for rectangular weir with 30 mm grain size bed under randomly bed configuration.

**Fig. 17:**
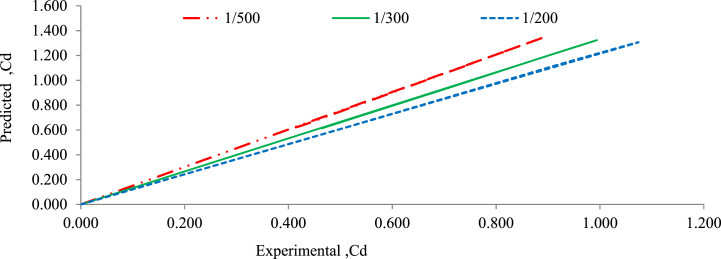
Validation of predicted and experimental values of coefficient of discharge for rectangular weir with 20 mm grain size bed and randomly bed configuration.

For rectangular weirs, Fig. 16, Fig. 17 show a good agreement among experimentally measured discharge coefficient results and data-predicted discharge coefficient results using the empirical model for 20 mm and 30 mm bed material size distribution and bed slope under randomly bed configuration. For actual and expected coefficient discharge values, validation for the suggested empirical models is shown in Equation (13) for experimental and predicted values [30,31].

Fig. 16, Fig. 17 show the experimentally obtained results and the data predicted by the empirical formula and they are in good agreement. In comparison to 30 mm grain size gravel bed material, the discharge coefficient for 20 mm grain size bed material increase as the bed slope changed from flatter to steeper. The validation of the discharge coefficient also demonstrated a high level of agreement between the experimental and projected values. With all bed material models and all bed slopes, the best fit of the relationships between experimental discharge coefficient and predicted discharge coefficient was obtained using the method of linear regression analysis; the typically R^2^ was well above 0.999 for both the grain size of gravel bed under all bed slopes.

The linear regression analysis performed on the relationship between the predicted and experimental discharge coefficients indicates a direct proportional relationship between these two coefficients. This finding indicates that as the predicted discharge coefficient increases, the observed discharge coefficient increases proportionally and linearly.

### 3.12 Experimentally validation of the proposed empirical model theoretical discharge Equation

The developed discharge algorithm is used to verify discharge accuracy by changing theoretical discharge values to obtain the actual discharge, whereas if Cd values are not constant, attempt was made to figure out the functional relationship. As a result, modifications were conducted by calculating geometric size and weir shape characteristics (K& n). The weir constant is determined experimentally using Equation (19) from the preceding general discussion section. Experiments are carried out to ascertain the constant for a specific weir. The corresponding discharges for the various heads over the crest are determined by measuring the amount of water discharged over the weir and is described by [88].

(19) $$Q=KH^{n}$$

By taking the logarithms of both sides of Equation 19

(20) LogQ=LogK+nLogH

When; H=1, LogH=0, then LogQ=LogK.Where; Log Q = value on y-axis, Log H = value on x-axis and Log k = y-intercept

(21) $$n=\frac{\mathrm{Log}\;Q-\text{LogK}}{\text{LogH}}$$

The values of K and n derived by the approach are substituted in Equation (19) to get the proposed weir's rating equation (equations (20), (21))). The results of each parameter and the corresponding rating equation were presented in Table 9 under both bed configurations.

**Table 9:** Calibrated Head –Discharge Rating Equation for Rectangular Weir with both bed configurations.

Weir type	Bed Slope	Test	Weir Rating Equations	Bed configuration
Rectangular Weir	1:500	1	$Q=0.026H^{\;0.71}$	Randomly
		2	$Q=0.053H^{0.970}$	
		3	$Q=0.096H^{1.212}$	Perpendicular to flow
		4	$Q=0.22H^{1.516}$	
	1:300	5	$Q=0.023H^{0.601}$	
		6	$Q=0.0128H^{0.505}$	Randomly
		7	$Q=0.072H^{1.096}$	
		8	$Q=0.216H^{1.495}$	Perpendicular to flow
	1:200	9	$Q=0.014H^{0.422}$	
		10	$Q=0.0125H^{0.474}$	Randomly
		11	$Q=0.038H^{0.886}$	Perpendicular to flow
		12	$Q=0.214H^{1.481}$	

Note: Q = discharge and H = depth of flow.

**Fig. 18:**
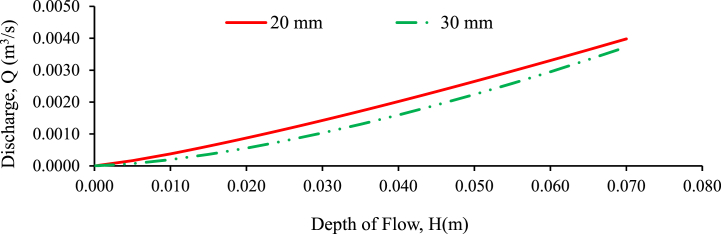
Experimentally validating proposed discharge equation for rectangular weir for 1:500 bed slope and different bed material under perpendicular to flow bed configuration.

**Fig. 19:**
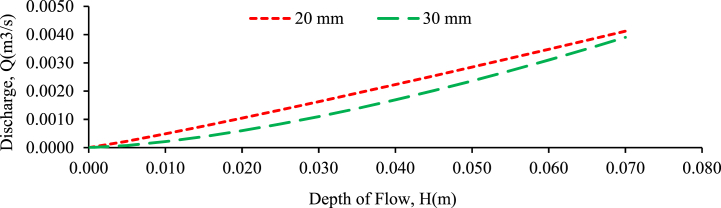
Experimentally validating proposed discharge equation for rectangular weir for 1:300 bed slope and different bed material under perpendicular to flow bed configuration.

**Fig. 20:**
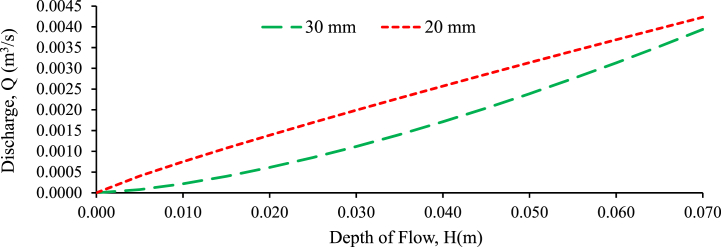
Experimentally validating proposed discharge equation for rectangular weir for 1:200 bed slope and different bed material under perpendicular to flow configuration.

The purpose of this experiment is to determine the relationship between the head-on weir and the consequent discharge Q over the rectangular weir in random and perpendicular to flow bed configurations. The discharge of the weir is determined on the coefficient of discharge as per the weir equation (Ahmed et al., 2017; [4]). The primary causes of inaccuracy to be handled in the check calibration of a measuring structure are [95,96], incorrect measurement of other geometric dimensions leads in an inaccuracy in the coefficient of discharge and flow depth estimation, incorrect crest width or flume throat measurements result in a systematic inaccuracy in the outflow, instruments of recording are unpredictable, incorrect gauge zero causing a systematic inaccuracy in head measurement and discharge. Fig. 18, Fig. 19, Fig. 20 shows the discharge versus upstream water head of regular rectangular weirs plotted under perpendicular to flow bed configuration for the bed slopes of 1:500, 1:300 and 1:200 respectively. As the figures show the head-discharge rating curve has good agreement between them because the correlation coefficient approaches one. The trend line of the graphs is linear, representing the discharge and head which has direct relationship between them. As the water level rose and the channel bed slope transitioned from steeper to flatter, the water flow rate increased. This trend was observed across both randomly arranged and perpendicularly-oriented bed configurations [2,3]. The head (stage) of flow increases, the discharge increases and vice versa in both bed configuration and grain size gravel bed samples [9,10]. The discharge and depth of flow (stage of flow) have higher relationship for 20 mm grain size gravel bed with the bed slope of 1:500, 1:300 and 1:200 compared to 30 mm grain size gravel bed model under randomly and perpendicular to flow bed configuration respectively.

A stronger relationship indicates a higher correlation between the variables, implying that changes in flow depth are more closely related to changes in discharge for the 20-mm grain-size gravel bed. This stronger relationship implies that, in this particular configuration, the depth of flow was a more reliable indicator or predictor of discharge than the 30 mm grain-size gravel bed.

## 4 Conclusion

According to the findings, flow circumstances, bed grain size, bed slope, bed structure, open channel features, and the presence of flow resistance all have an impact on the values of Manning's coefficient and discharge coefficient. Under all circumstances, a higher flow rate was associated with a lower value for Manning's coefficient. The flow depth and the flow resistance coefficient are found to be proportionate. Manning's n grows together with the flow depth. The value of the bed roughness indicates that the presence of roughness on the channel bed significantly affects the roughness, flow, and velocity characteristics of the open channel. The distribution of the bed, the depth of the flow, and the physical characteristics of the bed roughness itself all affect how rough the open channel is. The increasing trend of Manning's n values with decreasing depth indicates that, under both bed configurations with and without a weir—flow resistance increases throughout all bed slopes. The flow's Reynolds number is the main determinant of the resistance coefficient. The Manning roughness coefficient for fixed bed material was linearly proportional to tailgate water levels and inversely correlated with discharge, Froude, and Reynolds numbers. The discharge rises in tandem with the roughness coefficient. It is shown that 20 mm grain size gravel surfaces have a lower roughness coefficient and less of an inhibiting effect on water flow than 30 mm grain size gravel surfaces at a bed slope of 1:500. With and without a weir, the bed layouts are 1:300 and 1:200. The weir type influences discharge and establishes the ideal Cd values for rectangular weirs under various bed configurations, according to the testing results. Additionally, it is determined that, under a random bed arrangement, the actual coefficient of discharge over a rectangular weir is larger than that of a discharge perpendicular to the flow bed configuration. Under a random bed configuration of a 20 mm grain size gavel bed, the theoretical discharge value for a rectangular notch is between 0.39 and 0.79, 0.42–0.84, and 0.46–0.86; under a perpendicular bed form with a bed slope of 1:500, 1:300, and 1:200, respectively, it is between 0.27 and 0.64, 0.30–0.53, and 0.31–0.68. The discharge of flow via a rectangular weir is directly proportional to the discharge coefficient. In practical applications, the proposed model can be used to calculate the roughness coefficient for different water flow depths in the open channel.

## Data availability

All the data sets used to support the findings of the study are available in the supplementary file.

## Funding statement

No fund from any source has been received for this research work.

## CRediT authorship contribution statement

**Ayalkie Belete Amsie:** Writing – original draft, Data curation, Conceptualization. **Abebe Temesgen Ayalew:** Writing – review & editing, Methodology, Investigation, Formal analysis. **Zerihun Makayno Mada:** Writing – review & editing, Resources, Methodology. **Mekuanent Muluneh Finsa:** Writing – review & editing, Supervision, Resources.

## Declaration of competing interest

The authors declare that they have no known competing financial interests or personal relationships that could have appeared to influence the work reported in this paper.
